# Integrating mechanistic modelling with Bayesian optimisation: accelerated self-driving laboratories for RAFT polymerisation

**DOI:** 10.1039/d5dd00258c

**Published:** 2025-08-26

**Authors:** Clarissa. Y. P. Wilding, Richard. A. Bourne, Nicholas. J. Warren

**Affiliations:** a School of Chemical Process Engineering, University of Leeds Woodhouse Lane LS2 9JT UK C.Y.Wilding@leeds.ac.uk; b School of Chemical, Materials and Biological Engineering, University of Sheffield Mappin Street Sheffield S1 3JD UK nicholas.warren@sheffield.ac.uk

## Abstract

Discovery of sustainable, high-performing materials on timescales to meet societal needs is only going to be achieved with the assistance of artificial intelligence and machine learning. Herein, a Bayesian optimisation algorithm is trained using *in silico* reactions facilitated by a new mechanistic model for reversible addition fragmentation chain transfer polymerisation (RAFT). This subsequently informs experimental multi-objective self-optimisation of RAFT polymerisation using an automated polymerisation platform capable of measuring the critical algorithm objectives (monomer conversion and molecular weight distribution) online. The platform autonomously identifies the Pareto-front representing the trade-off between monomer conversion and molar mass dispersity with a reduced number of reactions compared to the equivalent fully experimental optimisation process. This model-informed AI approach provides opportunities for much more sustainable and efficient discovery of polymeric materials and provides a benchmark for other complex chemical systems.

## Abstract 2

Here, we have developed an accelerated self-driving laboratory that utilises a computationally inexpensive model to inform multi-objective Bayesian optimisation, to further minimise experimental overheads for precision RAFT polymerisation.

## Graphical abstract

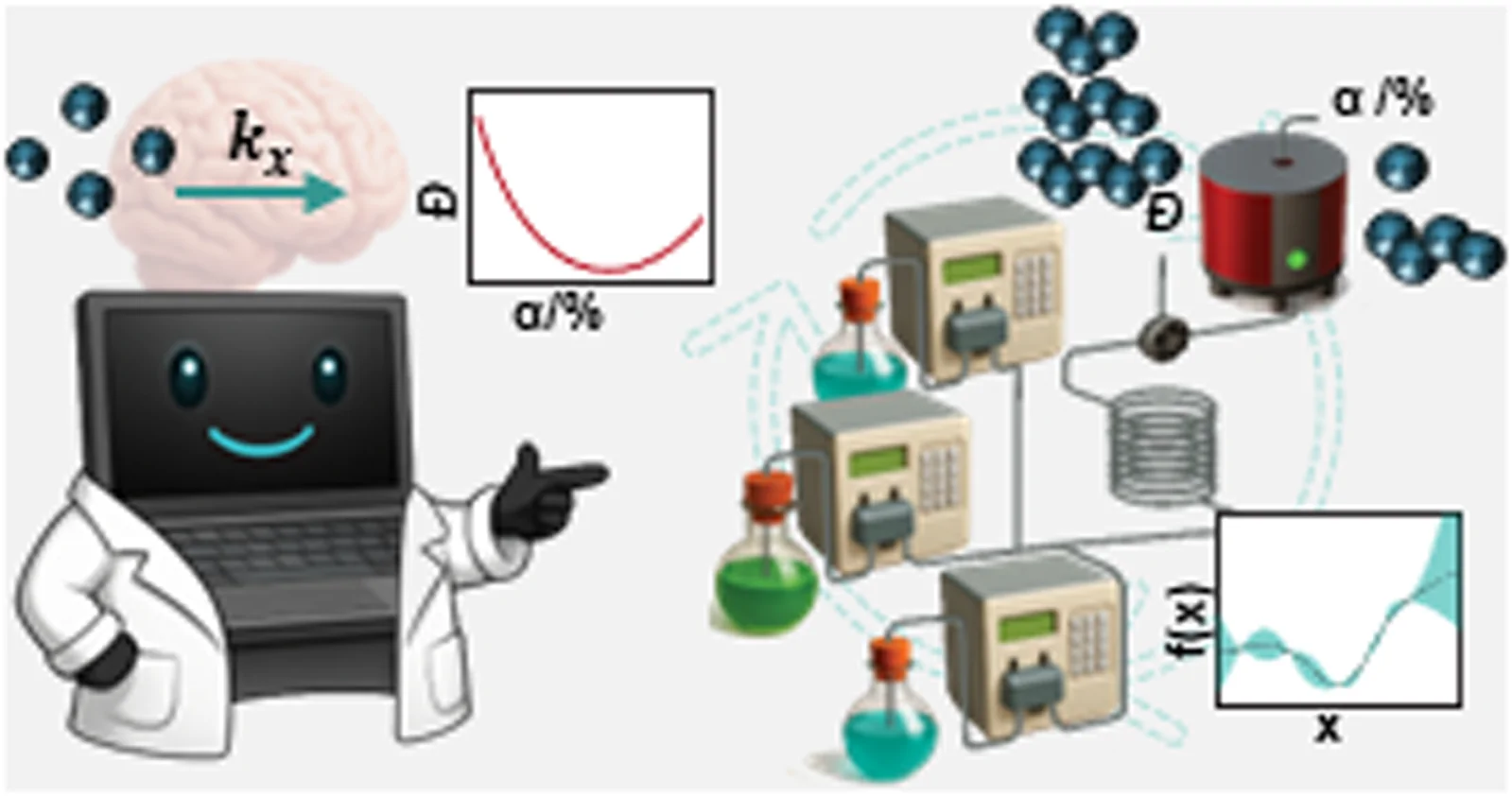

## Introduction

Autonomous experimental platforms are revolutionising the discovery of new chemistries and the rational design of novel materials.^1–3^ These platforms enable unprecedented high-throughput screening campaigns, and when integrated with advanced machine learning algorithms, they provide exponentially more efficient methods for exploring vast parameter spaces.^4–6^ Considering the limitless potential combinations of atoms in molecules, these cutting-edge approaches signify a paradigm shift in chemical synthesis, unlocking possibilities previously beyond our reach.

While small-molecule chemistry is inherently complex, polymer chemistry presents different challenges; scientists must also navigate the complexities of molecular weight distribution, monomer composition, and polymer architecture (*e.g.* block *vs.* statistical copolymers).^7^ These additional challenges make the implementation of AI (Artificially Intelligent) enabled platforms in polymer chemistry a formidable and intricate frontier.

Even though polymers play an immeasurable role in daily life from applications in drug delivery, to additives in nearly all liquid formulations, it is doubtful that the materials, and their manufacturing routes are truly optimised.^8^

Gaining a precise understanding of structure–property relationships in tandem with fine control over materials structure using AI enabled reactors has the potential to truly optimise material performance and unlock applications that are currently beyond our reach. Achieving these goals first requires the ability to optimise the synthetic process, which from the chemistry perspective has been achieved through much more accessible controlled polymerisation reactions.^9^ The most important class of these are reversible de-activation polymerisation (RDRP) techniques, including reversible addition fragmentation chain transfer (RAFT) polymerisation, atom transfer radical polymerisation (ATRP) and nitroxide mediated polymerisation (NMP).^10–12^ These chemistries have incrementally evolved since conception, including reduction in catalyst concentrations,^13^ removal of unwanted components (*e.g.* metals^14^ or sulfur^15^) and use of more efficient and sustainable photochemical,^16–18^ electrochemical^19,20^ and sonochemical^21^ initiation methods.

The optimisation of these processes still relies significantly on dated laboratory techniques, including one-factor at a time (OFAT)^22^ and design of experiments (DoE) approaches to screen conditions.^23–25^ These techniques can lead to local optima^22^ and increase experimental overheads of inherently complex reactions such as polymerisation.^22,26^ The polymer chemistry community is beginning to exploit an array of technologies to accelerate innovation.^27^ This includes developments in the areas of machine learning,^6,26^ reactor design,^25,28^ online monitoring,^29–31^ and computer control^32–35^ which have the potential to facilitate the next era of polymeric materials.

Automated experimental flow chemistry platforms have recently been applied to RAFT polymerisation, including the ability to facilitate programmable targeting of molecular weight and/or conversion, and to conduct automated kinetic screens.^33,36^ Each of these approaches required integration of gel permeation chromatography (GPC) and/or benchtop nuclear magnetic resonance spectroscopy (NMR) as measurement tools. Warren and co-workers also exemplified the first use of these for closed loop multi-objective Bayesian optimisation (BO) of RAFT polymerisation.^34^ Within this work, it was shown that the Thompson sampling by efficient multi-objective optimisation algorithm (TS-EMO), could efficiently map out a trade-off between monomer conversion (*α*%; measured by NMR) and molar mass dispersity (*Đ*; measured by GPC) in a 2D parameter space where residence time and temperature were the input variables. Further examples included its use for polymerisation-induced self-assembly^37^ and aqueous emulsion polymerisation.^38^ Importantly, these examples report experimental platforms that required no prior knowledge of the chemistry, other than defining the bounds of reaction space.

In polymer chemistry, since kinetics are relatively well studied, and rate coefficients for the important steps are well reported in the literature,^39–42^ the availability of such information is beginning to reduce reliance on real experiments, with so-called *in silico* experiments providing a wealth of information which can be used for better defining parameter space. For example, Kandelhard *et al.*^25^ used commercially available modelling software (PREDICI and COMSOL) to optimise different reactors and conversion *in silico* which were then successfully validated using benchtop NMR. The caveat here was that it required decisions to be made by a trained human operator despite there often being an array of models to predict reaction outcomes. Several models for predicting the kinetics and molar mass dispersity of RDRP systems have been reported,^43–45^ including a combined kinetic and dispersity model for RAFT polymerisation. This relies on input of known propagation, chain-transfer, and termination constants, *k*_p_, *k*_tr_, and *k*_t_ for specific monomers and has been made available as a software package to facilitate *in silico* RAFT polymerisation. Furthermore, it allows for augmenting the effects of residence time distribution for modelling RAFT polymerisation in a flow reactor. This provided the optimal approach for validation through dissipating exotherms (typically observed in batch) which can cause unwanted rate acceleration.^45^ The emergence of these technologies has now set the scene for a model informed approach to conduct more sustainable machine learning directed polymer synthesis.

Herein, we demonstrate the first example of a pre-informed self-optimising polymerisation platform capable of exploring 3D parameter space facilitated by *in silico* modelling. Reaction optimisation, specifically multi-objective problems, can be considered a ‘black-box’ function as the objective function is unknown, complex or expensive to evaluate, meaning only a surrogate model based on input variable data and output analytical data is feasible to guide the optimisation.^46,47^ As with our previous work,^45^ all aspects of a closed-loop reactor platform were interfaced with the BO algorithm TS-EMO to enable the reciprocal exchange of data and control over conditions. TS-EMO was selected for its ability to outperform several multi-objective optimisation algorithms; including, Pareto efficient global optimisation (ParEGO), non-sorting genetic algorithm ii (NSGA-ii) and expected improvement matrix efficient global optimisation (EIM-EGO).^48,49^ In this work, three input variables (rather than two) define 3D reaction parameter space (residence time, temperature and initiator concentration), and the option to conduct and include *in silico* experiments to inform regions of interest was programmed. In both the fully experimental and model informed cases, the ability to map out the trade-off between low molar mass dispersity (*Đ*) and high conversion was characterised.

**Fig. 1:**
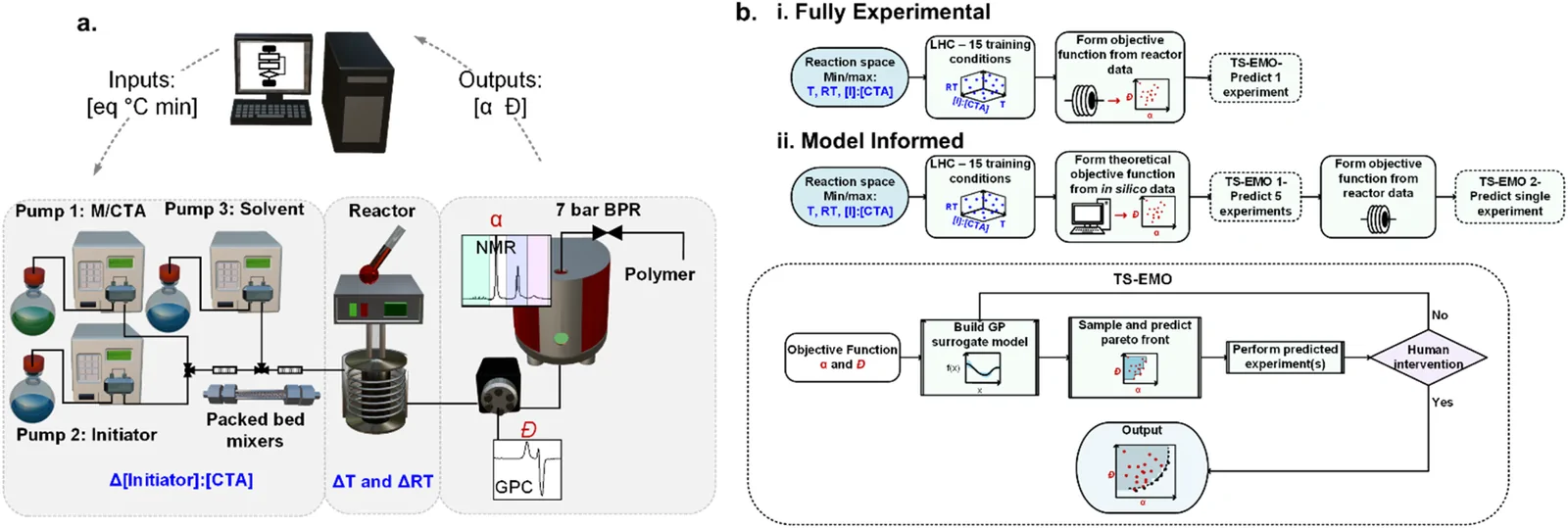
(a) General schematic of autonomous reactor platform used in this work, showing the reagent reservoirs, heated coil, and orthogonal analytical instrumentation in the form of at-line GPC and inline NMR. A back pressure regulator (BPR) is used to maintain pressure and enable temperatures greater than the solvent boiling point. (b) The two optimisation protocols used in this paper for the self-optimisation of dimethyl acrylamide and *n*-butylacrylate: (i) fully experimental which operates exclusively on experimental *α* and *Đ* data from the reactor platform and (ii) model-informed which simulates the *α* and *Đ* for the initial LHC experiments and predicts an initial search space.

A computer-controlled reactor platform was constructed, enabling an autonomous feedback loop by varying input variables (temperature, residence time, and initiator concentration) and analysing output data (monomer conversion and molar mass dispersity). This advanced setup included three pumps, two packed bed mixers, a temperature-controlled tubular flow reactor, a benchtop NMR for monitoring monomer conversion, a GPC setup for obtaining the molecular weight distribution. This latter process also required and an in-line switching valve for sampling the reaction solution emerging from the reactor, which was then diluted and passed though the GPC. A schematic of the platform can be observed in Fig. 1a.

This autonomy was enabled through the development of a bespoke graphical user interface which was configured to operate in two ways (Fig. 1b): the first operates in a wholly empirical manner – where the algorithm learns exclusively from experimental data (termed “fully experimental”). The second operates in a model-informed manner, where the algorithm is trained using *in silico* data obtained using our previously developed model for RAFT polymerisation,^45^ (termed “model-informed”). As demonstrated in Fig. 1b(i) and (ii), respectively. Both optimisation campaigns use training data obtained by selection of experiments in the defined parameter space using LHC sampling, forming an objective function containing 15 experiments. In the model-informed approach, a preliminary objective function is generated from simulated *α* and *Đ* data obtained from the *in silico* LHC, which is then used to predict 5 experiments using an initial iteration of TS-EMO^50^ (TS-EMO 1). As the model is not a fully analytical function of the reaction/reactor, the model-informed approach can still be considered black-box. Upon executing these 5 experiments, and the results are used to construct a new experimentally obtained objective function. Subsequent, iterations of TS-EMO (TS-EMO 2) then suggested single experiments which were evaluated experimentally and used to update the experimentally obtained objective function. This approach provides two layers of training data which considers both the chemistry, and an understanding of how the reactor configuration affects the process (*e.g.* RTDs and heat transfer). These approaches provide the main TSEMO campaign with the most reliable training dataset for exploration and exploitation.

## Results and discussion

### Three variable self-optimisation of the RAFT polymerisation of dimethylacrylamide

**Scheme 1:**
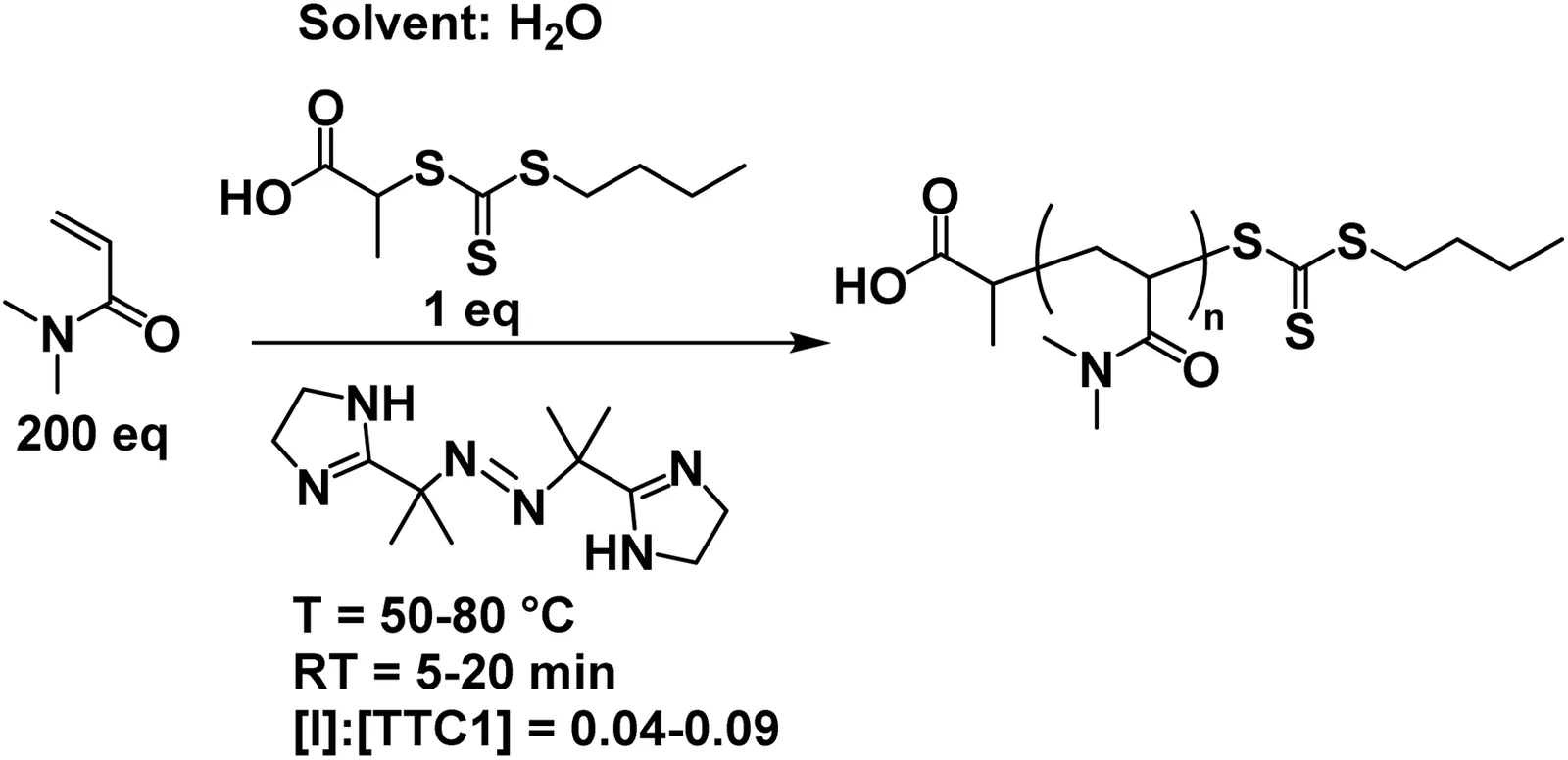
Reaction scheme for the aqueous RAFT polymerisation of *N*,*N*-dimethyl acrylamide using trithiocarbonate (TTC1) in the presence of VA-044 initiator. Optimisation limits for temperature, residence time and initiator concentration are shown in the scheme.

**Fig. 2:**
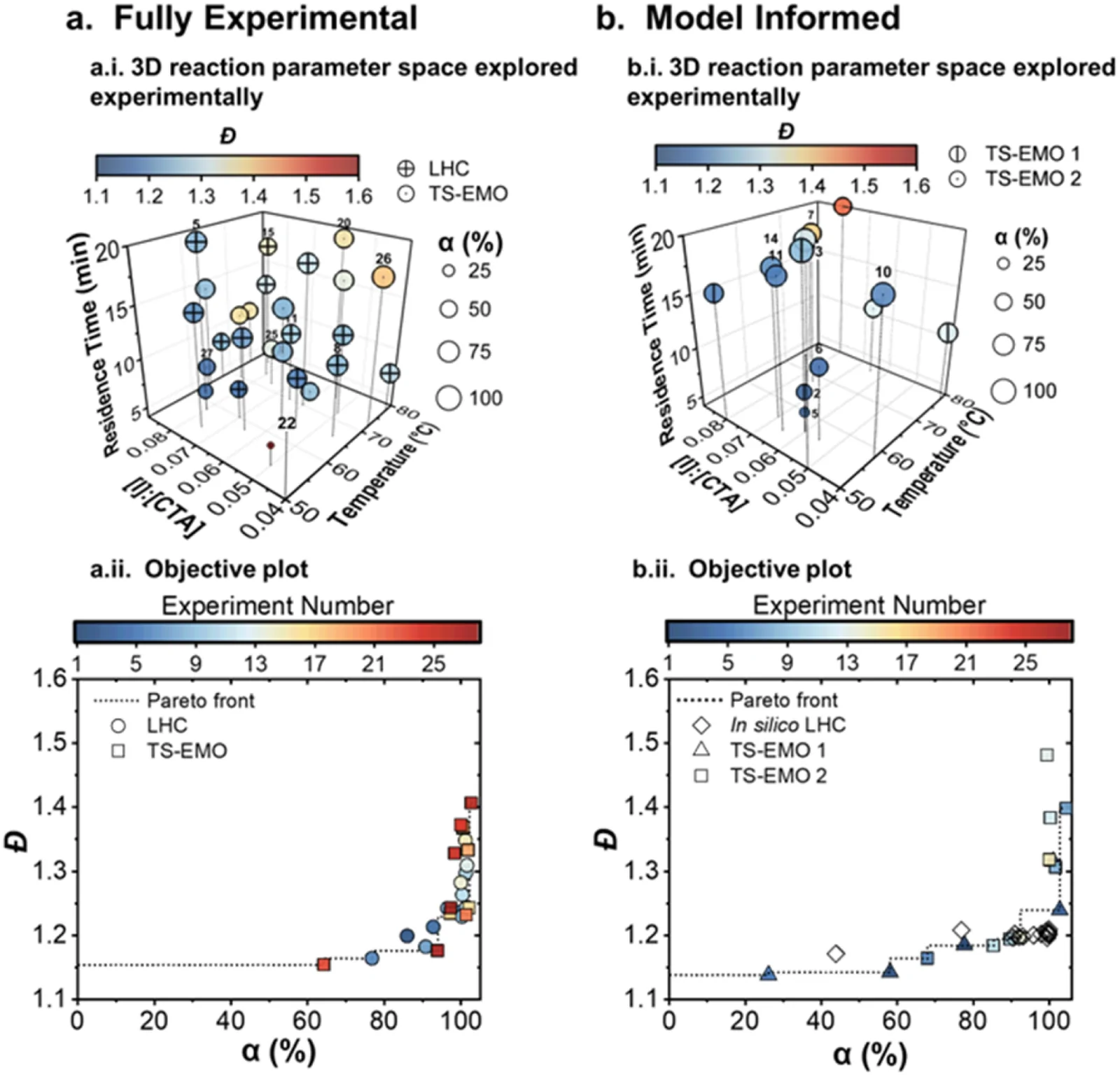
Optimisation of the conversion (*α*) and dispersity (*Đ*) for the polymerisations of DMAm in the presence of TTC1 using VA-044 as the initiator comparing fully experimental approach (a) with model informed approach (b). 3D bubble plots with three input variables shown on the axes and experimentally obtained *α* (size) and *Đ* (colour), are demonstrated in (a.i.) and (b.i.), for the fully experimental and model informed approach, respectively. The experiment number corresponding to the Pareto front are labelled. Objective plots showing trade-off in *Đ* and *α* optimisation for the fully experimental (a.ii.) and model-informed (b.ii.) approaches. The progression of the experimental data collected is illustrated by the colour of the data point.

A 3D parameter space was initially explored for the aqueous RAFT polymerisation of dimethylacrylamide (DMAm) (Scheme 1) where initial training experiments defined by LHC were used to guide the TSEMO algorithm, which despite the additional input variable (initiator concentration), provided valuable insights into the effects of each input parameter on the outcome of the reaction. As expected, the highest monomer conversion was achieved at elevated temperature, high initiator concentration and long residence times (Fig. 2a and b(i)). Importantly, the algorithm mapped out the trade-off between conversion and molar mass dispersity, which gradually broadened under more forceful conditions. This is a trade-off which the TS-EMO algorithm defines relatively well at high conversion, with a tendency to “over-explore” this area. This may be due to the algorithm anticipating the biggest hypervolume gains by conducting these experiments (see Fig. S11 for the hypervolume convergence plots). There was a reduced tendency to explore areas of lower *Đ* because it anticipates these gains are likely to be small based on the surrogate model.

The subsequent model-informed experiments involved the *in silico* simulations using conditions defined by LHC sampling – see black diamonds in Fig. 2b(ii). This data was obtained in approximately 15 s using a standard laboratory PC (see SI for specifications), compared to the fully experimental platform which took 561 minutes to conduct the physical set of training experiments. This simulated data was then used to generate a preliminary objective function approximation that informed the first iteration of the TSEMO algorithm which generated 5 conditions for physical experimentation on the flow platform.^51–53^ Once these were completed, the data was fed into a second iteration of TSEMO that would suggest single experiments that could gain hypervolume. The model informed approach mapped the Pareto front more effectively and efficiently (Fig. 2b), including determination of more non-dominated solutions at lower conversion and lower dispersity compared to the fully experimental optimisation (Fig. 2a). When there was no visible qualitative improvement in the Pareto front the optimisation was terminated. On plotting the hypervolume obtained (Fig. S11) it is clear after 15 experiments the total hypervolume exceeds that obtained during the fully experimental method (which conducted 22 experiments).

The accuracy of the model is further validated in these experiments, where the *in silico* data overlaps the experimental data. It can be ascertained from the plots in Fig. 2b that there is a positive correlation between the three control variables (temperature, initiator concentration and residence time) on the conversion. Furthermore, more reaction space corresponding to areas of low dispersity was investigated by the model informed platform compared to the experimental platform shown in Fig. 2a. When considering the condition selection, it is clear the model informed platform selects more conditions at lower temperatures which is likely a result of the model identifying this as a region of interest pertaining to a steady supply of radicals and reduction in terminative events.

As a result, the model-informed platform identified more points with the lowest dispersity such as point 2 in Fig. 2b(ii) where GPC indicates a *Đ* of 1.14 whilst NMR reports a conversion of 58%. The conditions for this experiment involved an 8 min residence time at 56 °C using 0.06 eq. of initiator (relative to CTA). The closest non-dominated solution in the fully experimental plot was found after 6 min at 58 °C using 0.08 eq. of initiator – where 64% conversion was achieved with a *Đ* of 1.15. There is a huge difference in the number of experiments required to find these low *Đ* points, the fully experimental platform found the lowest *Đ* in 22 experiments compared to just 2 in the model informed optimisation. This is in the first iteration of the TSEMO algorithm for the model-informed platform, which is the initial experimental exploration, so we can assume that this is not a fortuitous result as the first iteration learns directly from the model. In contrast to the fully experimental platform, the model-informed platform finds conditions that lead to lower *Đ*, thus widening the exploration of reaction space.

Within this optimisation algorithm, a set of hyperparameters generated comprising the mean and covariance are used to generate a surrogate mathematical model. The covariance function has an inbuilt noise parameter associated (*σ*^2^) which allows the algorithm to handle experimental data. Conveniently, *σ*^2^ can be used to determine how important each output parameter is for controlling the optimisation process, where a lower *σ*^2^ indicates more effective choice of conditions for obtaining non-dominated solutions. Consequently, it determines whether there are truly benefits in using the model informed approach over the fully experimental approach.

The significantly lower noise hyperparameters (see Table 1) associated with the model-informed optimisation (*σ*_MI_2) compared to the experimental optimisation (*σ*_exp_^2^) demonstrate that the model directed platform is much more efficient than the fully experimental approach, with identified solutions leading to the Pareto front being identified in 40% fewer experiments than the fully experimental optimisation. This is a 58% reduction in experiment time and a saving of 270 mL of reagent solution demonstrating a step-change in the efficiency of chemical synthesis optimisation (Table 2).

**Table 1:** Calculated *σ*^2^ hyperparameters for the fully experimental (*σ*_exp_2) and model-informed (*σ*_MI_^2^) self-optimisation of aqueous RAFT polymerisation of *N*,*N*-dimethyl acrylamide using TTC1 in the presence of VA-044 initiator. Larger values indicate more noise and less effective exploration of parameter space. GP refers to gaussian process model

Hyperparameters	GP1 (dispersity)	GP2 (conversion)
*σ*_exp_^2^	0.32	0.46
*σ*_MI_^2^	0.04	0.09

**Table 2:** Experimental requirements to determine Pareto fronts optimal conditions for the fully experimental *vs.* model-informed platform for the RAFT polymerisation of dimethylacrylamide

	Fully experimental optimisation	Model-informed optimisation
Total number of experiments	28	13
Number of training experiments	15	5
Volume of solution (mL)	450	180
Time taken to map the Pareto front (h)	17	7

### Three variable self-optimisation of the RAFT polymerisation of butyl acrylate

**Scheme 2:**
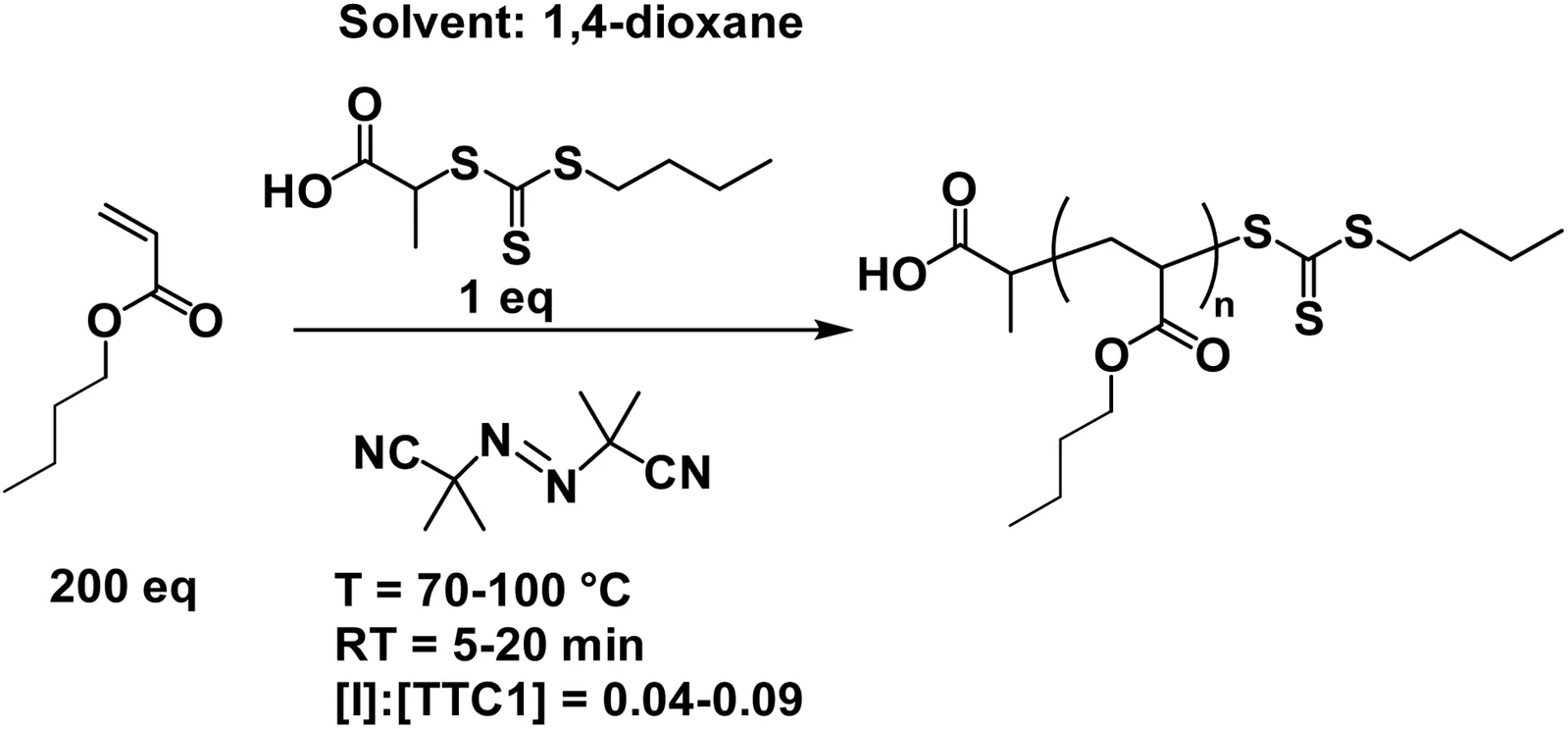
Reaction scheme for the RAFT polymerisation of *n*-butylacrylate using TTC1 in the presence of AIBN initiator in dioxane, optimisation limits for temperature, residence time and initiator concentration are shown in the scheme.

Further validation of the approach was pursued for the RAFT solution polymerisation of butyl acrylate in dioxane (Scheme 2). This polymerisation is complicated by numerous side-reactions under more extreme conditions at high conversion (such as formation of mid-chain radicals, cross-termination, and beta scission).^54^ These side reactions which can cause broader molecular weight distributions and higher *Đ* values are not included within the derivation of the model.^45^ As a result, the expectation was that there would be a significant deviation between *in silico* predictions and experimental observations.

**Fig. 3:**
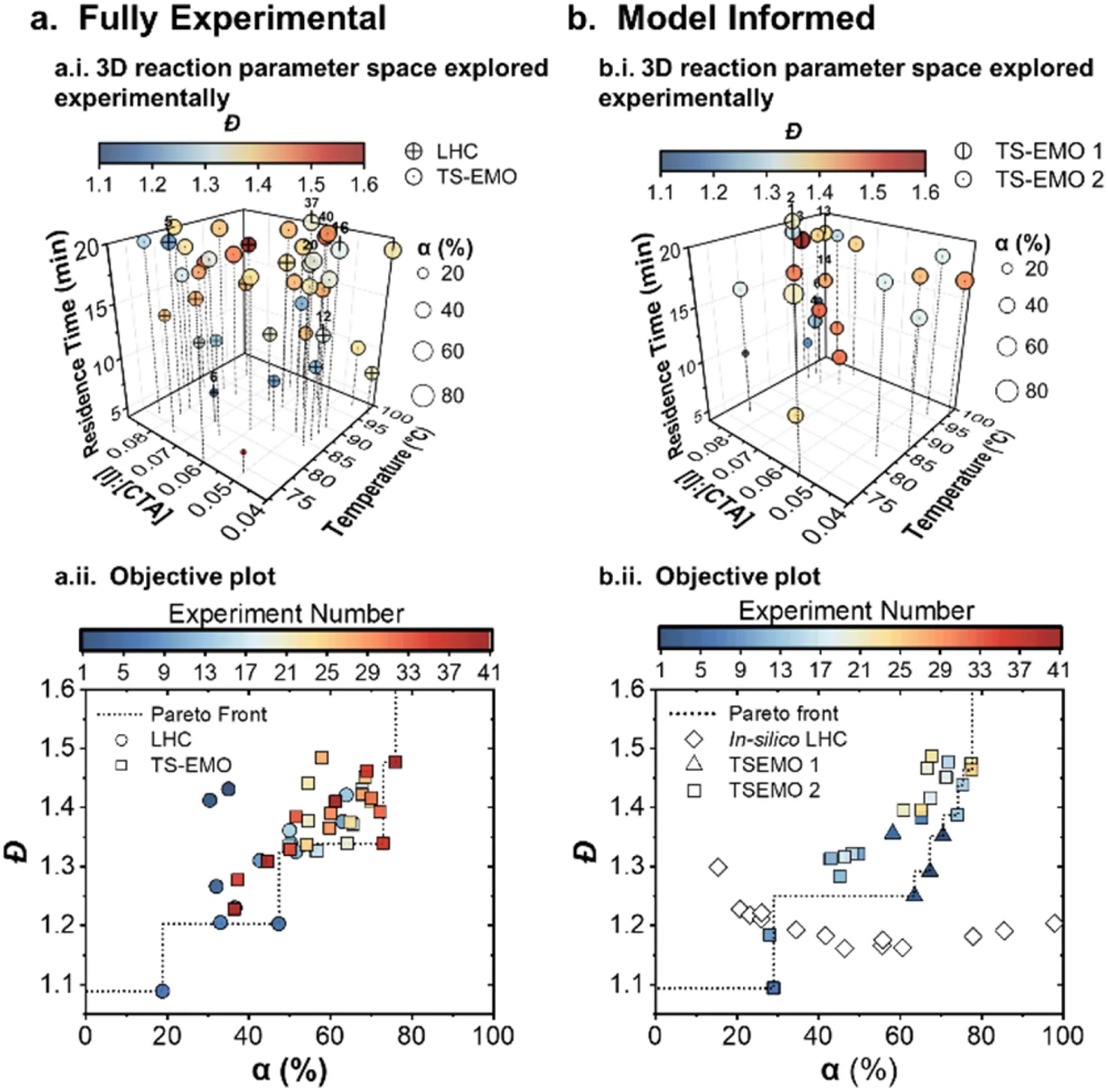
Optimisation data for the polymerisations of butylacrylate in the presence of trithiocarbonates using AIBN as the initiator comparing fully experimental approach (a) with model informed approach (b). 3D bubble plots with three input variables shown on the axes and experimentally obtained *Đ* (size) and *Đ* (colour), are demonstrated in (a.i.) and (b.i.), for the fully experimental and model informed approach, respectively. The experiment number corresponding to the Pareto front are labelled. Objective plots showing trade-off in *Đ* and *α* optimisation for the fully experimental (a.ii.) and model-informed (b.ii.) approaches. The progression of the experimental data collected is illustrated by the colour of the data point.

During the fully experimental optimisation, there is a steep increase in molar mass dispersity and an inability to attain conversion higher than 80%. The Pareto front is also relatively poorly defined, with only four points contributing to hypervolume gains (see Fig. 3a(ii)). The non-dominated solution with the lowest molar mass dispersity *Đ* = 1.09; point 6 in Fig. 3a(i) was obtained at a low monomer conversion of 17%. The non-dominated solution with the highest conversion is obtained after 19 min at 100 °C using 0.06 eq. of initiator to obtain a conversion of 76% but a broad molecular weight distribution (*Đ* = 1.47).

For the model-informed experiment, marginal improvements are observed with respect to optimal samples (either high conversion or low dispersity) but notably, the Pareto front is better defined at higher conversion. In this case, the reaction yielding the polymer with the lowest dispersity has around a 10% improvement in monomer conversion (Fig. 3b), whereas the highest conversion non-dominated solution provides a negligible improvement in molar mass dispersity (1.46 *vs.* 1.47).

The simulated reactions, which assume only initiation, propagation, chain-transfer, and termination, show minimal up-turn in dispersity at high conversion (Fig. 3b(ii); black diamonds). This contrasts with experimental results, where there is a much larger up-turn. This directly reflects the added complications for acrylate polymerization, including slower propagation from mid-chain radicals.^54^ This example highlights the importance of the three-stage optimisation process: initial *in silico* experiments followed by two iterations of TSEMO. The *in silico* experiments can simulate the “ideal” chemistry, while the first TSEMO iteration optimises for the real system, providing crucial real-world data which is influenced by side reactions in addition to reactor-specific phenomena including fluid dynamics (manifesting as a broader RTD) and non-ideal heat transfer. This data contributes to improving the relatively poor initial surrogate model providing a better basis for the second TSEMO campaign. This methodology demonstrably reduced experimental overheads by over 35% compared to the black box study.

Once again, quantitative comparison (see Table 3) for the optimisations indicates the model-informed approach is more effective, where *σ*_MI_2 is smaller than *σ*_exp_2. However, the difference in this case is less drastic, which is a likely result of the more complex chemistry. Nevertheless, it demonstrates that implementation of a model that predicts the best-case scenario can be used as the foundation to a thorough optimisation of the real system. This results once again in the Pareto front mapping being achieved more efficiently, using fewer experiments, less time, and materials (Table 4).

**Table 3:** Calculated *σ*^2^ hyperparameters for the fully experimental (*σ*_exp_2) and model-assisted (*σ*_MI_2) self-optimisation of RAFT polymerisation of *n*-butylacrylate in dioxane using TTC1 in the presence of AIBN initiator. Larger values indicate more noise and less effective exploration of parameter space

Hyperparameters	GP1 (dispersity)	GP2 (conversion)
*σ*_exp_2	0.33	0.63
*σ*_MI_2	0.21	0.31

**Table 4:** Experimental requirements to determine Pareto fronts optimal conditions for the fully experimental *vs.* model informed platform for the RAFT polymerisation of butyl acrylate

	Fully experimental optimisation	Model-informed optimisation
Number of experiments	42	26
Number of training experiments	15	5
The volume of solution (mL)	714	442
Time taken to map the Pareto front (h)	36	22

## Conclusion

To conclude, a step change in the ability to optimise RAFT polymerisation has been achieved through development of a novel reactor to expand parameter space, and implementation of a mechanistic model-guided multi-objective self-optimisation algorithm. The use of a computationally inexpensive model significantly reduced the time, amounts of reagents and cost of the self-optimisation process for DMAm and *n*BuA. The model has been shown to reduce the number of dominated solutions and explore more of the Pareto front where low dispersity is observed. Enabling polymer chemists to explore the objective space in the region most desired *i.e.*, high conversion/low dispersity. Not-only is this more effective but the cost of running an optimisation is reduced by up to 35% through reduced reagent consumption and platform time. The efficiency of the model directed optimisation of the RAFT polymerisation of *n*BuA also provides the potential to enable steady-state self-optimisation of monomers that are more challenging to synthesize/low yielding. It also provides insights into how similar optimisation frameworks can be developed for other types of synthesis (*i.e.*, small molecules) where a model is available to further reduce experimental overheads which has important implications regarding product development rate, sustainability and economic cost.

## Author contributions

CYPW contributed to the conceptualisation, software/model development, formal analysis, experimentation, visualisation, writing, review and editing. NJW conceptualised the project, acquired funding, contributed to writing, review and editing. RAB contributed to conceptualisation, writing review and editing.

## Conflicts of interest

There are no conflicts to declare.

## Supplementary Material

DD-004-D5DD00258C-s001

## Data Availability

Full reactor details, model, algorithm schemes and data tables can be found in the SI. Annotated source code for implementing the model informed optimisation campaigns have been archived in the Zenodo repository: https://doi.org/10.5281/zenodo.16909796. See DOI: https://doi.org/10.1039/d5dd00258c.
