# Supplementary material for: Integrating mechanistic modelling with Bayesian optimisation: accelerated self-driving laboratories for RAFT polymerisation
Source: Digit Discov. 2025 Aug 26;4(10):2797–803. doi: 10.1039/d5dd00258c (PMC13580203; doi:10.1039/d5dd00258c)
Supplement: Supplementary file 1 [file DD-004-D5DD00258C-s001.pdf]

Supplementary Information (SI) for Digital Discovery.
This journal is © The Royal Society of Chemistry 2025

Supplementary information

Integrating Mechanistic Modelling with Bayesian Optimisation: Accelerated Self-Driving Laboratories for RAFT Polymerisation

Clarissa. Y. P Wilding*[a] Richard A. Bourne[a] and Nicholas J. Warren*[a,b]

[a] School of Chemical and Process Engineering,
University of Leeds,
Woodhouse Lane, LS2 9J

[b] School of Chemical, Materials and Biological Engineering,
University of Sheffield,
Mappin Street, Sheffield, S1 3JD, UK

1. Table of Contents

2. Materials **3**
3. Reactor platform **3**
a. Reactor Construction 3
Computer specifications 5
Analysis 6
Calculations of conversion from 60 MHz NMR 6
Gel Permeation Chromatography 8
b. Algorithms 9
Thompson Sampling by Efficient Multi-objective Optimisation (TSEMO)[2] 9
Model 11
Modified algorithm used for model-informed platform. 14
4. RAFT Polymerisation of dimethylacrylamide **16**
Data 17
Performance Metric 19
5. RAFT Polymerisation of n-butyl acrylate **20**
Data 22
Performance Metric 25

1. Materials

N,N-Dimethyl acrylamide 99 % containing 500 ppm of monomethyl ether hydroquinone inhibitor (DMAm, CAS – 2680-03-7), n-butyl acrylate 99 % containing 10-60 ppm of monomethyl ether hydroquinone inhibitor (nBuA, CAS – 141-32-2) and 2,2'-Azobis(isobutyrontirile) 98 % (AIBN, CAS – 78-67-1) were purchased from Sigma Aldrich.
4,4'-Azobis[2-(2-imidazolin-2-yl)propane]dichloride 97 % (VA-044, CAS - 27776-21-2) was purchased from Wako Chemicals. 1,4-Dioxane 99+ %, ACS reagent, stabilised (CAS - 123-91-1) was purchased from Fisher Scientific. 2-(Butylthiocarbonothioylthio)propanoic acid (TTC1, CAS – 480436-46-2) was purchased from Boron Molecular. These were used without purification.

2. Reactor platform

a. Reactor Construction

The reactions conducted in this manuscript were performed in a continuous flow reactor comprising three Jasco high performance liquid chromatography (HPLC) pumps PU-980/1580, directed the reaction solution through a 5 mL (outer diameter (OD) = 1/16", inner diameter (ID)=0.762 mm) 304 stainless steel tubular reactor controlled by a Eurotherm (model 3216) temperature controller fitted with 2 Elmatic Max K cartridge heaters. The individual streams were mixed inline by directing through standard Swagelok T-pieces (1/16") followed by a packed bed mixer unit made from a 30 mm of stainless-steel tubing (OD = 1/8", ID= 2.1 mm) packed with 40 mesh SiO_2 beads plugged at either end with glass wool.

Monomer conversion was measured by in-line Magritek Spinsolve 60 Ultra benchtop nuclear magnetic resonance (NMR) operating at a frequency of 60 MHz, programmed to run a shim on sample before each run.
A glass guide, the diameter of a standard NMR tube, was used to thread 1/8" perfluoroalkoxy alkane (PFA) tubing through to ensure that the tube remained stationary in the NMR, as illustrated in Figure S 1. a. Molecular weight data (M_n and $Đ$) recorded by extracting a sample via a 2 position Vici 6-port switching valve placed in series, which directed the sample (100 µL) through a custom built at-line gel permeation chromatography (GPC) instrument. This comprised a Jasco HPLC -PU 980 operating at 2 mL min^{-1} consisting of a 5 µm Guard column

(equilibrated to 35 °C to avoid further polymerization) followed by an Agilent Rapide M column connected in series with a Knauer 2301 refractive index detector. Values were calculated based on a series of near-monodisperse poly (methyl methacrylate) (PMMA) standards with molecular weights (M_p) between 800 to 2,200,000 g mol^{-1}. A cartridge back pressure regulator rated to 5 bar, was positioned after the Rapide M guard column to ensure a stable flow rate throughout the GPC. The columns were heated to 35 °C to reduce the viscosity effects of the eluent, dimethyl formamide (DMF), the heating system is pictured in Figure S 1. b.

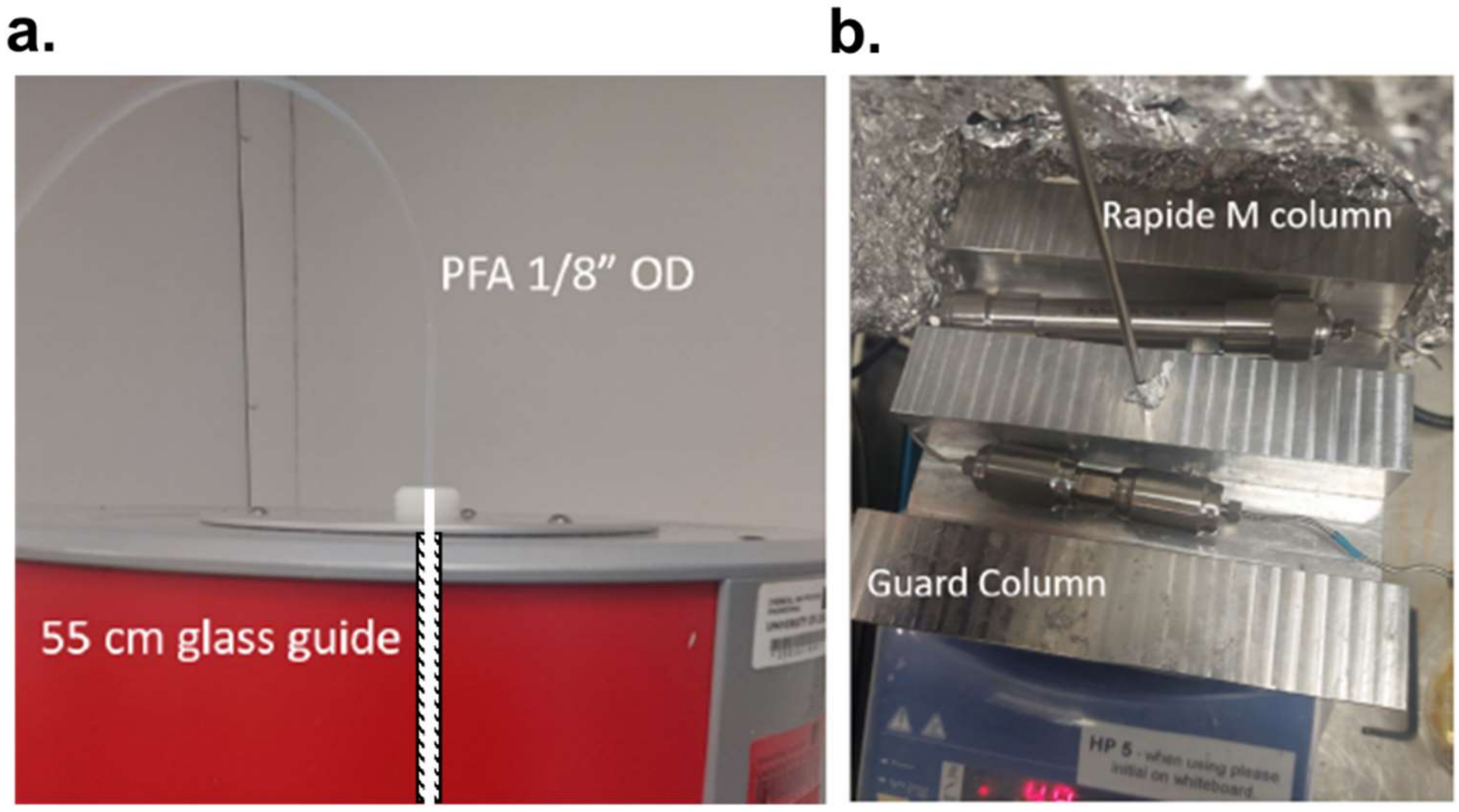

Figure S 1. *(a) NMR analysis set-up showing how a 55 cm glass tube is used to guide the PFA tubing through the NMR to ensure that the tube remains stationary. (b) GPC column set up for heating both columns on a Asynt hotplate using a bespoke aluminium heating block to heat the columns to 35 °C and monitor the temperature using the thermocouple.*

Each pump contained a serial communications board that was connected to the pc using the RS232 protocol via a male 25 pin to female 9 pin serial cable and a male 9 pin to male USB (FTDI chip). Modbus protocol was used to communicate with the Eurotherm temperature controller, which was connected via a female 9 pin serial to female 9 pin and then to a Male USB (FTDI). The VICI switching valve loop and actuator was controlled by RS232 communications using 3 pin female adaptor cable to 9 pin DB9. Refractive index was collected by the Knauer RI detector connected using the analog output to a Pico Technology Pico logger (TC-08). The NMR was connected by USB directly to the PC, to enable external control of the NMR using the Spin solve software a TCP/IP connection was established. To accommodate all USBs a female USB bus was used.

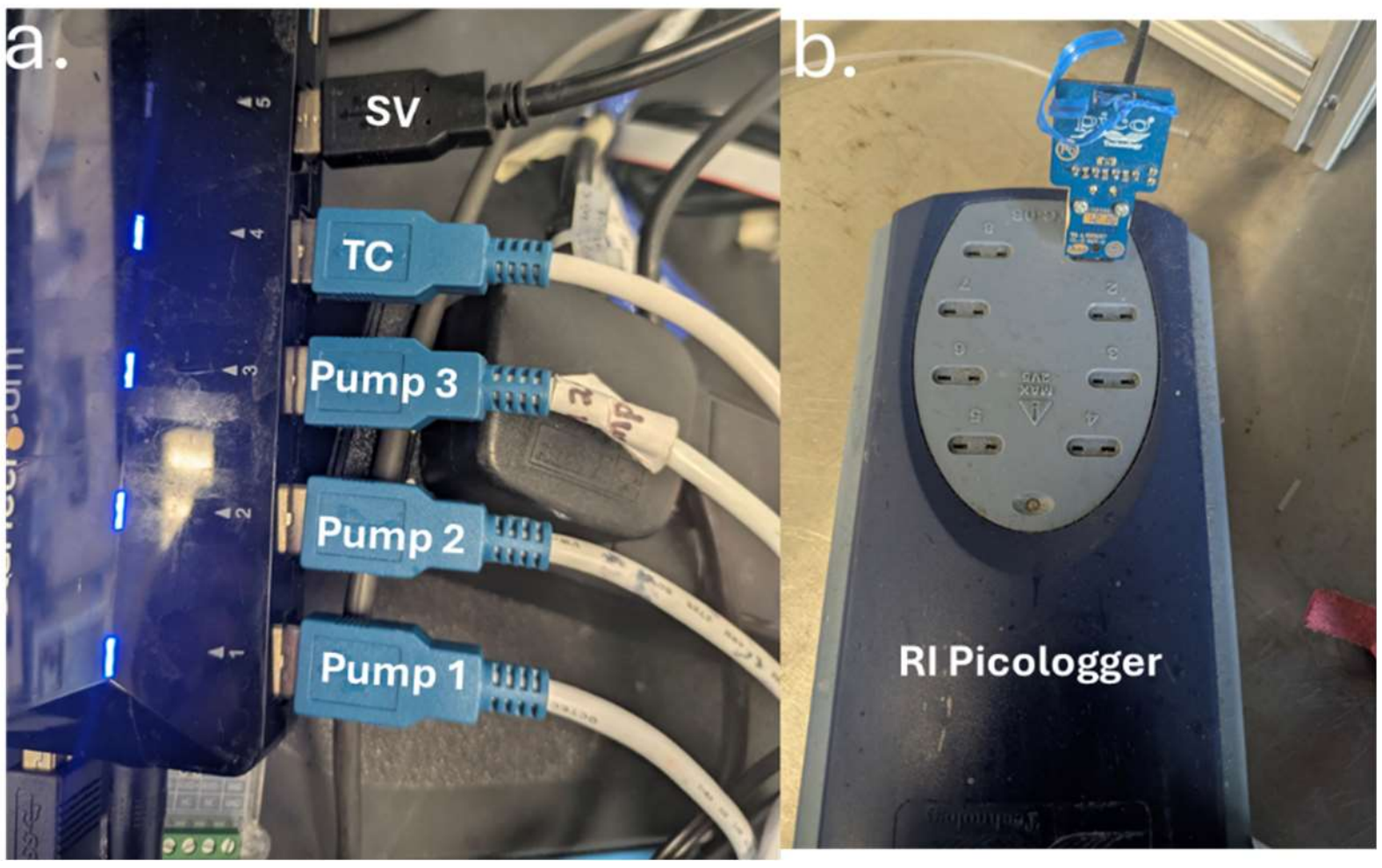

Figure S 2*.(a) Photograph of USB bus used in the reactor platform and (b) data logger used to obtain the RI output.*

Computer specifications

A standard laboratory PC was used for both simulations and optimisation. Intel(R)Core i5-7400T @2.40 GHz, 2401 MHz, 4 Cores, 4 Logical processors, 32 GB RAM

a.

b.

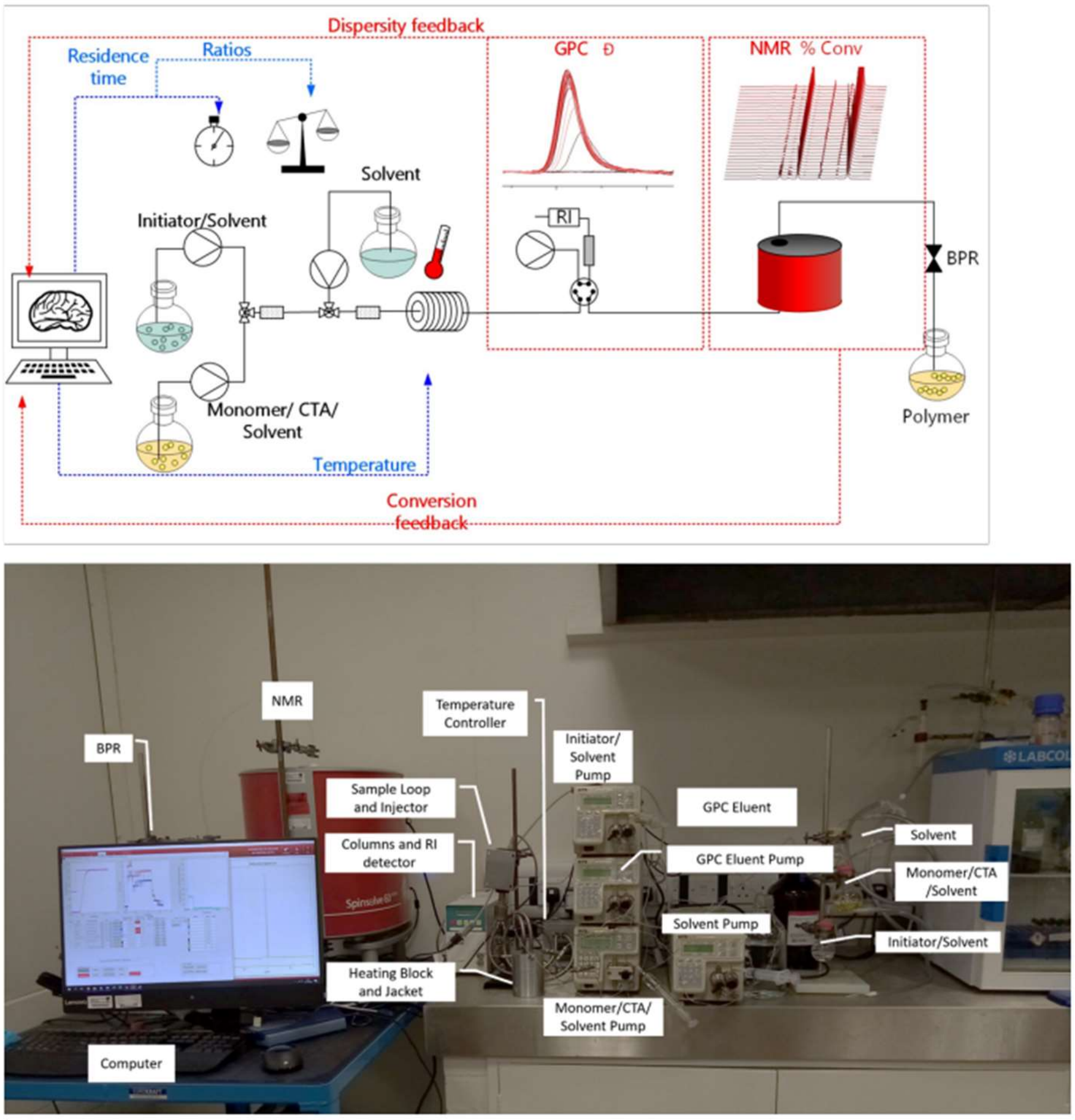

Figure S 3. *(a) Schematic of the reactor used in this paper for the optimisation of conversion (α) monitored by NMR, dispersity (Đ) measured by GPC. Reaction occurs in a 5 mL stainless steel reactor coiled around an aluminium heating block fitted with cartridge heaters. The initiator concentration is controlled by the relative pump flowrates, temperature is controlled by a temperature controller and the residence time is controlled by the overall flow rates of the pumps. A Zaiput BPR pressurised to 7 bar was also used to enable low boiling point solvents to be used such as methanol. (b) Photograph of the platform showing the 3 reagent pumps, GPC pump, GPC set up and NMR.*

Analysis

Calculations of conversion from 60 MHz NMR

The general method for NMR analysis was programmed to conduct a pre-saturation solvent suppression. This consisted of a 1 s -65 dB saturation pulse at 4.79 ppm for the aqueous RAFT polymerisation of dimethylacrylamide, and 3.53 ppm for the RAFT polymerisation of

butyl acrylate in 1,4-dioxane. The spectral width was set to 5 kHz, an acquisition time 6.4 s, repetition time of 10 s and 2 scans.

Details of how conversion is calculated from benchtop NMR data can be found in Knox et al. [1]Conversion was calculated by comparing the integral of the vinyl peaks (5.5 -7.0 ppm) and comparing it to the polymer backbone region (1.0-2.5 ppm) whilst accounting for pendant groups.

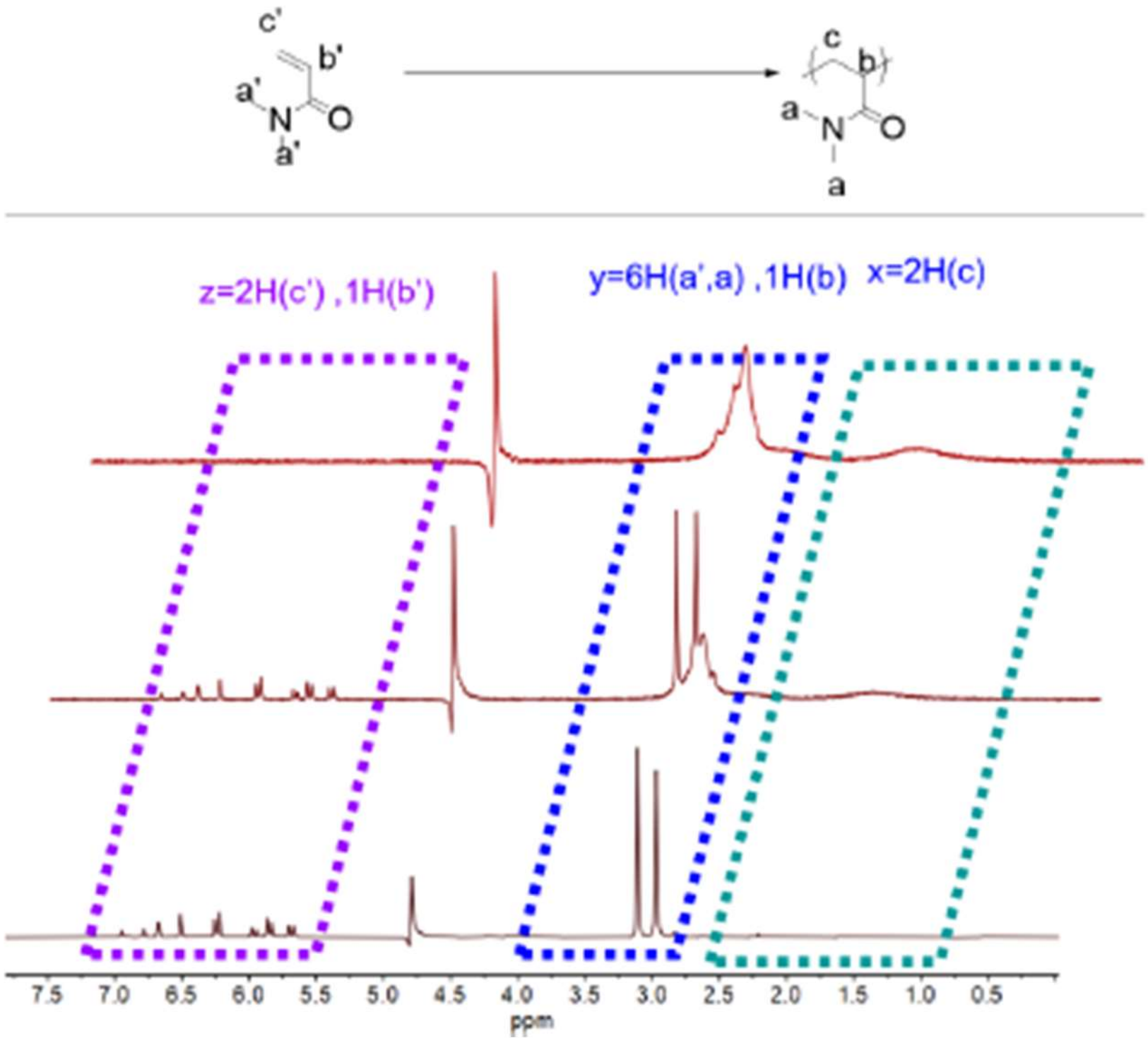

***Figure S 4**. An example of how conversion is calculated from 60 MHz benchtop NMR for the polymerisation of dimethylacrylamide.*

Where α is monomer conversion, z is the integral of the vinyl region containing 3 protons, y is the shared pendant groups containing 6 protons and 1 proton on polymer backbone in position b. Integral x, is the 2 protons in proton environment c on the polymer backbone.

$$\alpha = 1 - \frac{2z}{x+y} \qquad \textit{Eq S 1}$$

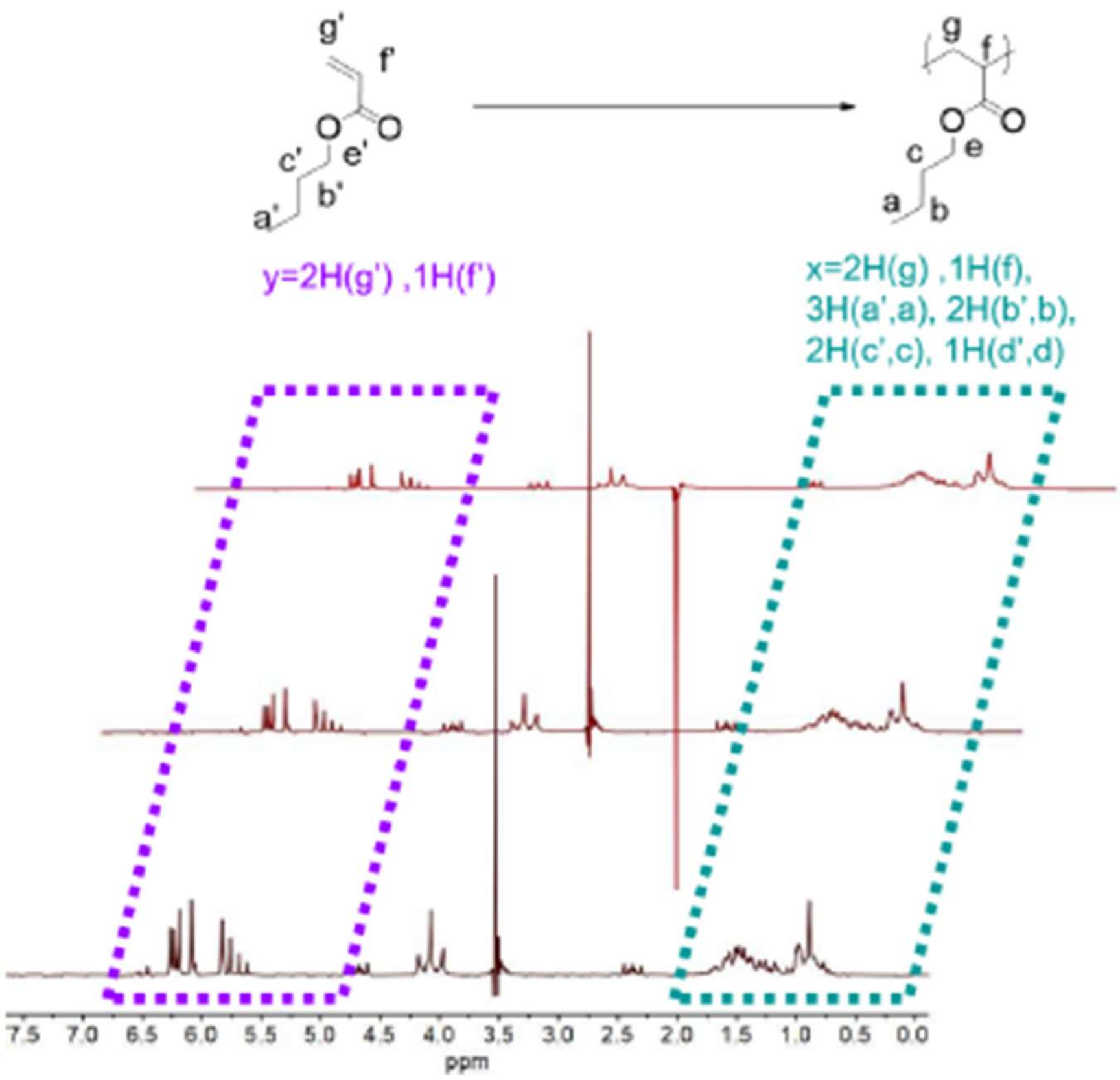

Figure S 5. *An example of how conversion is calculated from 60 MHz benchtop NMR for the polymerisation of butylacrylate.*

In this example, y represents the 2 protons in the vinyl region, whilst x, represents the protons in the polymer backbone and the pendant groups on both the monomer and polymer. This is subsequently normalised.

$$\alpha = 1 - \frac{10x}{3(x+y)} \qquad \textit{Eq S 2}$$

Gel Permeation Chromatography

For the aqueous RAFT polymerisation of DMAm, dimethyl formamide containing lithium bromide (0.1 w/w %) was selected as the eluent. The columns were heated to 35 °C and calibrated using the PMMA standards. For the RAFT polymerisation of nBuA, tetrahydrofuran treated with triethylamine (1 w/w %) and BHT (0.05 w/w %) was selected as the solvent at room temperature and calibrated using the PMMA standards.

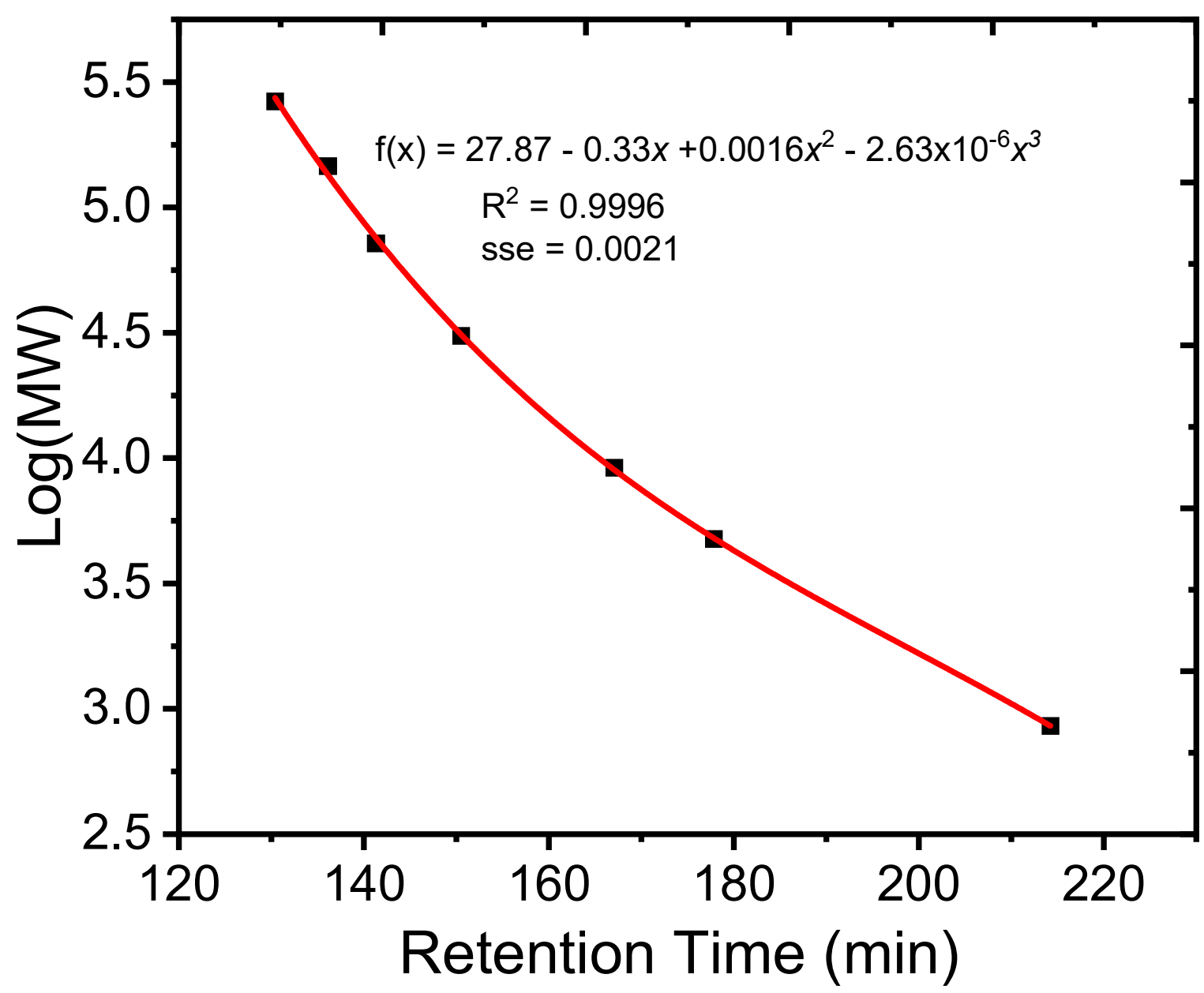

Figure S 6*. Example calibration curve for GPC analysis protocol.*

b. Algorithms

Thompson Sampling by Efficient Multi-objective Optimisation (TSEMO)[2]

The fully experimental implementation of TS-EMO is illustrated in **Figure S 7**. The algorithm was initialized by defining the bounds of reaction space (minimum and maximum values for temperature, residence time and [I]:[CTA] ratio) and the step limits for these variables. These can be found in **Table S 2** and **Table S 6**. This enables a Latin hypercube sampling algorithm to identify pseudo randomized experiments that span the reaction space[3–5]. These experiments were conducted by the reactor platform and the output data for *Đ and conversion* and subsequently used to form an objective function.

TSEMO makes use of Gaussian processes (GPs) as surrogate models which contain both a mean function, $m(\mathrm{x})$ and a covariance function, $k(\mathrm{x}, \mathrm{x}')$.

$$f(x) \sim GP\big(m(x), k(x, x')\big) \qquad \textit{Eq S 3}$$

TSEMO aims to solve the multi-objective optimisation process described in Eq S 4.

$$minimise_{x\in\chi\subseteq\mathbb{R}^d}\ \boldsymbol{G}(\mathrm{x}) = [g_1(\mathrm{x}), g_2(\mathrm{x}), \dots, g_m(\mathrm{x})] \qquad \textit{Eq S 4}$$

Eq S 4, describe the minimization of $\boldsymbol{G}$, which is a vector containing, m, scalar black-box objective functions, $g_i(\mathrm{x})$. Assuming that the objective functions are continuous, and can be evaluated at x, representing a query point, in domain χ and can handle noisy data that is unbiased.

The input vector, X^i, are scaled between 0 and 1, and updated with each iteration, i.

$$X^i = \begin{Bmatrix} x_{[I]:[CTA]_1} & x_{T_1} & x_{t_1} \\ \vdots & \vdots & \vdots \\ x_{[I]:[CTA]_i} & x_{T_i} & x_{t_i} \end{Bmatrix} \qquad \text{Eq S 5}$$

The response vectors, Y_j are also scaled between 0 mean and the unit variance, and updated with each iteration, where j =1, …, m, m is the number of outputs.

$$Y_\alpha^i = \begin{Bmatrix} y_{\alpha_1} \\ \vdots \\ y_{\alpha_i} \end{Bmatrix},\ Y_Đ^i = \begin{Bmatrix} y_{Đ_1} \\ \vdots \\ y_{Đ_i} \end{Bmatrix} \qquad \text{Eq S 6}$$

For each response output an independent GP using Matern kernel 1/2 is trained.

$$f_\alpha(x) = GP_\alpha^i(m^i, k^i | X^i, Y_\alpha^i) \qquad \text{Eq S 7}$$

$$f_Đ(x) = GP_Đ^i(m^i, k^i | X^i, Y_Đ^i) \qquad \textit{Eq S 8}$$

Once these are formed, the algorithm uses spectral sampling on each of the GPs in Eq S 7 and Eq S 8, to evaluate 4000 points. The algorithm then identifies a set of pareto optimal solutions using a non-dominated sorting genetic algorithm (NSGA-ii) which sorts the predictions based on, here the population size was set to default (100), the pareto optimal solutions (this is set to 1 per iteration) are selected based upon the expected hypervolume improvement. Once selected these are evaluated and added to the current data set.

Hypervolume is a performance criteria used to determine the pareto optimal conditions and is defined as an m-dimensional (m is the number of objectives) Lebesque measure of dominated space with respect to a reference point. [2]

$$HV(\mathbb{P}, R) = \lambda_m\left(U_{p\in\mathbb{P}}[p, R]\right) \quad \text{Eq S 9}$$

Where $HV(\mathbb{P}, R)$ is the hypervolume of the pareto front, $\mathbb{P}$,with respect to a reference point, R.

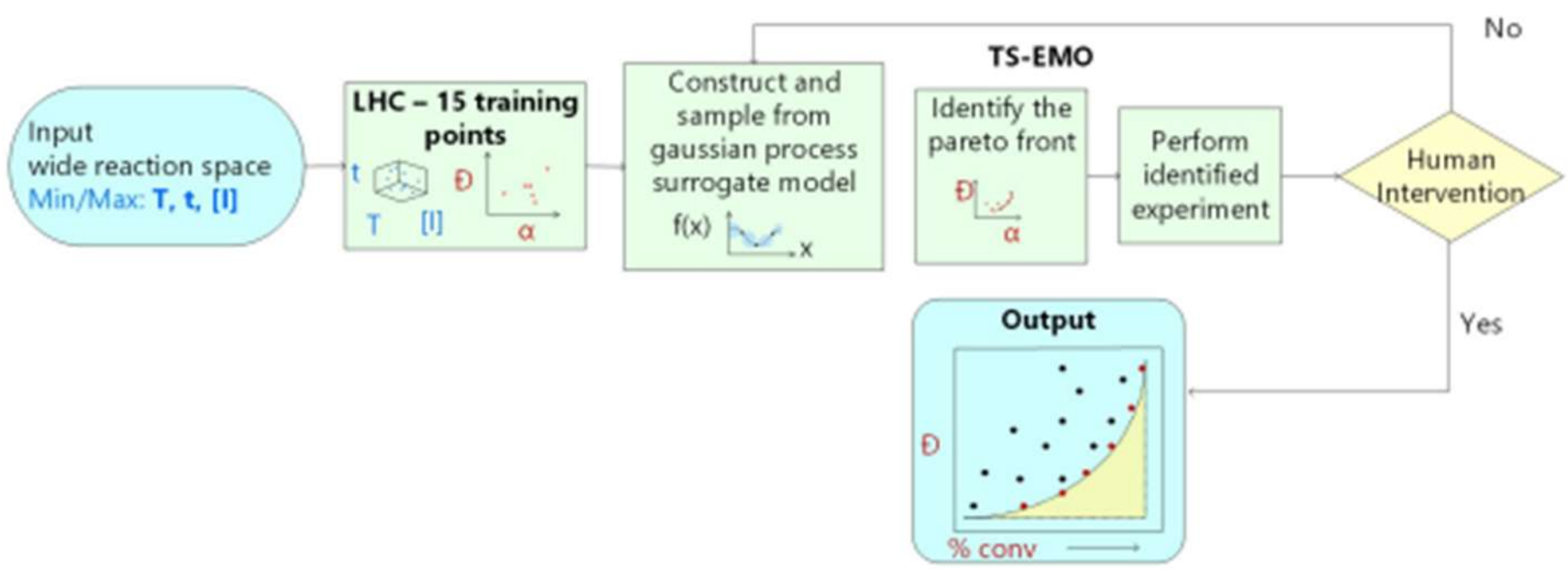

Figure S 7. *Flow chart of how the TS-EMO works for the fully experimental platform.*

Model

The model used to inform the algorithm is taken from Wilding et al.[6] Heat transfer and mixing were assumed to be ideal, and temperature dependence was accounted for using Arrhenius equation and steady state kinetics were assumed at each residence time.

The monomer conversion was calculated using an analytically solved mechanistic model that predicts the change monomer concentration over time assuming that monomers only end up in polymers and that the reaction is at steady state.

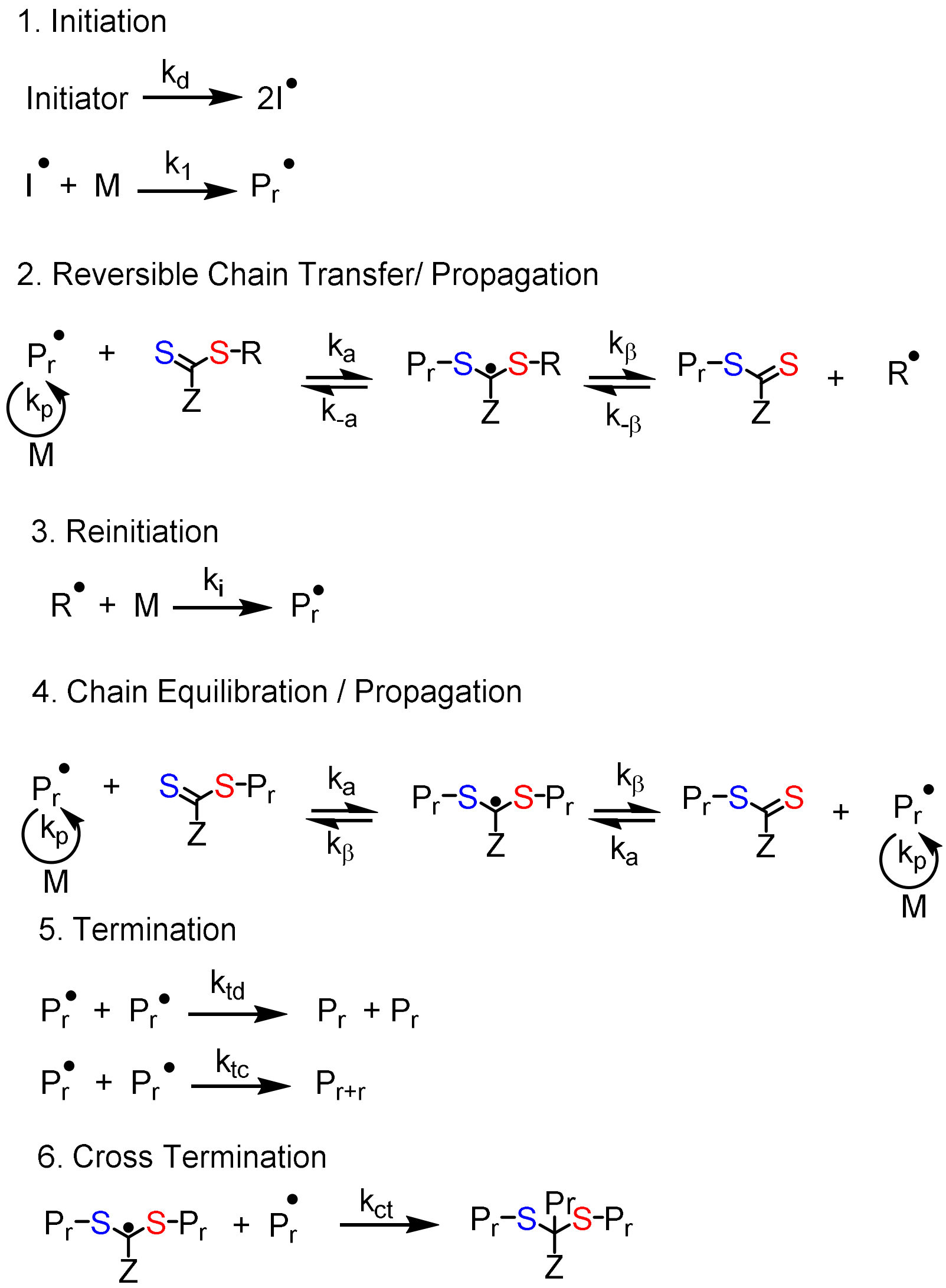

Figure S 8. *Mechanism of RAFT showing initiation, propagation, termination(disproportionation/combination), chain transfer (addition/fragmentation) and cross termination and the relative governing rate constants* k_d *,* $k_p, k_{td}, k_{tc}, k_a, k_\beta$ *and* k_{ct}*. In this instance, like free-radical polymerisation, initiation occurs when the azo initiator (I) undergoes thermal decomposition to generate 2 initiator radicals (I·) subsequently reacts with monomers (M) to form propagating radical species (*P_r*·). Propagating radicals can undergo further reaction with monomers to form longer chain radicals in a process called propagation. These chain radicals can undergo disproportionation (H transferred from one chain radical species to another forming two dead polymers) or recombination (two chain species combine to form a larger chain) to form polymer chains (*P_r*). In RAFT, chain transfer agents are introduced to reversibly capture the propagating chains via the radical adduct intermediate, to control the chain-length, and the molar mass dispersity. Reinitiation can occur when the R group radical*

expelled after the pre-equilibrium reacts with monomer to form another Propagating radical species. In some cases, the radical adduct intermediate in the chain-transfer steps can also form a covalent bond with another propagating radical, giving a 3-armed polymer species.

Table S 1 *Table of reaction steps and respective rate equations. (i) Initiation, (ii) propagation, (iii) pre-equilibrium, (iv) re-initiation, (v) main-equilibrium, (vi) termination, and (vii) cross-termination (formation of 3-armed species)*

Step	Rate Equations	#
$I_2 \xrightarrow{fk_d} 2I\cdot$	$\frac{d[I]}{dt} = -2fk_d[I]$	i
$M + P_r \xrightarrow{k_p} P_r$	$\frac{d[M]}{dt} = -k_p[M][P_r\ \cdot]$	ii
$P_r + CTA \underset{k_{-a}}{\overset{k_a}{\leftrightarrow}} CTA_a \underset{k_\beta}{\overset{k_\beta}{\leftrightarrow}} CTA + R$	$\frac{d[P_r]}{dt} = r_1 + k_\beta[CTA_a] - k_a[P_r\cdot][CTA] - 2k_t[P_r\cdot]^2 - k_{ct}[P_r\cdot][CTA_a]$	iii
$R + M \xrightarrow{k_i} P_r$	$\frac{d[CTA]}{dt} = k_\beta[CTA_a] - k_a[P_r\cdot][CTA]$	iv
$P_r + CTA \underset{k_{-a}}{\overset{k_a}{\leftrightarrow}} CTA_a \underset{k_\beta}{\overset{k_\beta}{\leftrightarrow}} CTA + P_r$	$\frac{d[CTA_a]}{dt} = k_a[P_r\cdot][CTA] - k_\beta[CTA_a] - k_{ct}[P_r\cdot][CTA_a]$	v
$P_r + P_r \xrightarrow{k_{td}} P_r$	$\frac{d[P]}{dt} = k_{td}[P_r\cdot]^2$	vi
$P_r + CTA_a \xrightarrow{k_{ct}} P$	$\frac{d[P']}{dt} = k_{ct}[P_r\cdot][CTA_a]$	vii

Experimentally, the reactor platform extracts monomer conversion using NMR, so a model based on work by Wang et al.[7] was used to model the monomer conversion , described by Eq S 10, which can be modelled by solving (ii) in **Table S 1**. This is conducted using literature

rate coefficients for initiator decomposition (k_d), propagation (k_p), addition (k_a), fragmentation (k_β), termination by disproportionation (k_t) and chain transfer to adduct intermediate (k_{ct}).[6]

$$[P_r] = \frac{\sqrt{2fk_d[I]_0 e^{-k_d t}}}{\left(\frac{k_t k_\beta}{k_{ct} k_a [CTA]} + 2\right)} \quad \text{Eq S 10}$$

$$\frac{d[M]}{dt} = -k_p[M]\left(\frac{\sqrt{2fk_d[I]_0 e^{-k_d t}}}{\left(\frac{k_t k_\beta}{k_{ct} k_a [CTA]} + 2\right)}\right) \quad \text{Eq S 11}$$

$$\alpha = 1 - \frac{[M]_t}{[M]_0} \quad \text{Eq S 12}$$

The monomer concentration was then elucidated using a numerical ODE solver in MATLAB on Eq S 11 and the conversion was predicted using Eq S *12*.

The Dispersity was calculated using an explicit model of the molar mass dispersity as a function of the conversion accounting for residence time distribution as derived in Wilding *et al.*[6] Here, chain transfer to the RAFT agent/macro-RAFT agent is given by k_{tr}. The model is an equation that accounts for chain growth and terminative events as described in **Table S 1**.

$$Đ = 1 + \frac{[\mathrm{CTA}]_0}{[\mathrm{M}]_0 \alpha} + \frac{k_p}{k_{tr}}\left(\frac{2}{\alpha} - 1\right) + \frac{k_t}{4k_p[\mathrm{CTA}]_0}\sqrt{\frac{r_R}{2k_t}}\alpha \quad \text{Eq S 13}$$

Modified algorithm used for model-informed platform.

In the case of the model informed platform the initial experiments were conducted in-silico. The initial LHC was conducted from this data, enabling an initial GP to be constructed for each objective, as expressed in Eq S 7 and Eq S 8. The algorithm identified 5 predicted pareto optimal solutions, which were then conducted experimentally. Once evaluated as a batch of

experiments, the GPs were overridden to only be constructed based on experimental data. Subsequent iterations were programmed to only evaluate a single pareto optimal experiment, as demonstrated in **Figure S 9**.

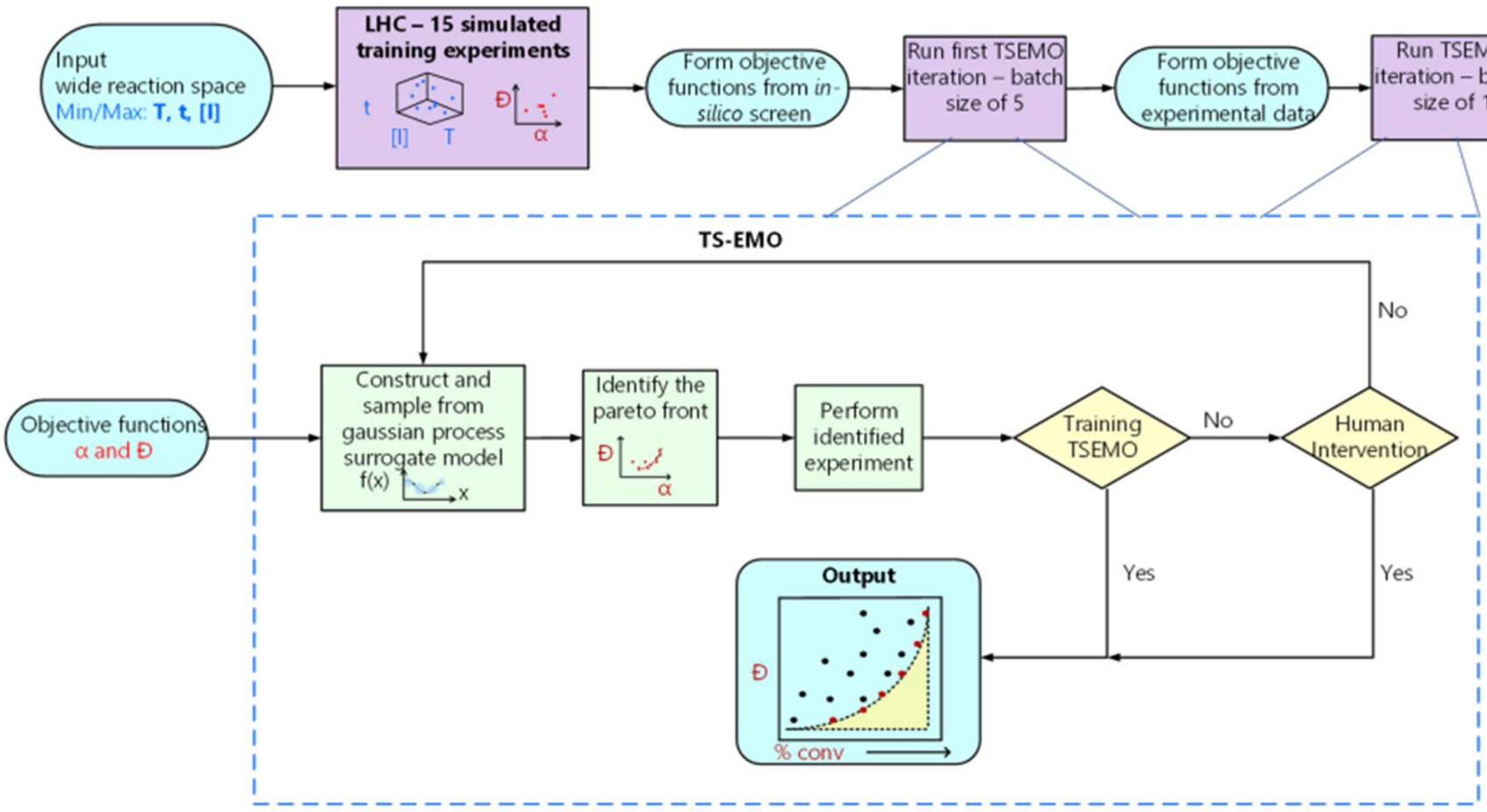

Figure S 9. *Flow diagram of how the algorithm operates with using an in-silico model to assist in defining reaction space of interest.*

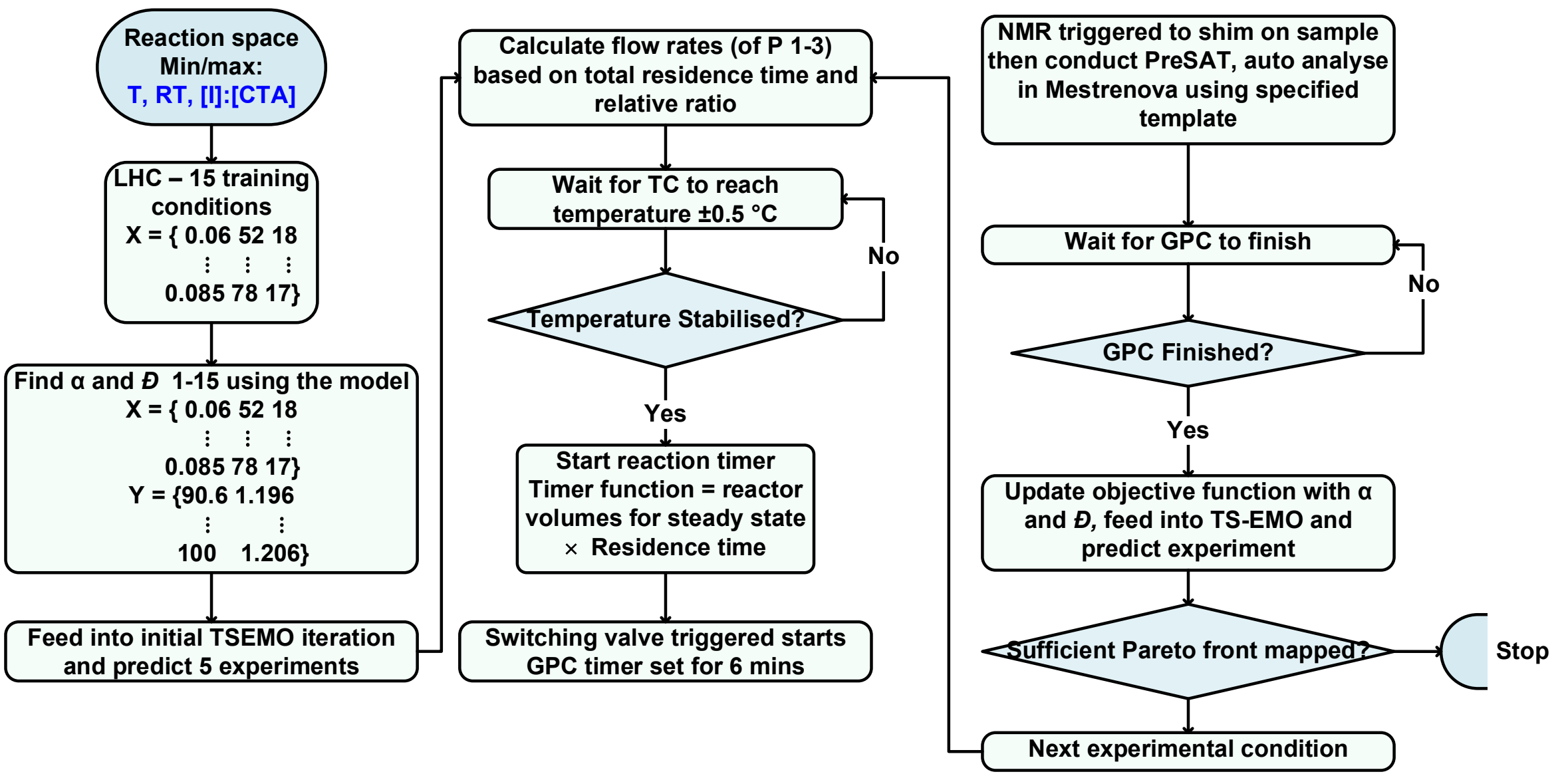

Figure S 10. Flow chart showing the how the model informed algorithm is used and decision making within the reactor platform

3. RAFT Polymerisation of dimethylacrylamide

To a solution of DMAm (250 g, 2.52 mol) in water (169.1 g), TTC1 (3.00 g, 0.013 mol) was added. A separate flask was charged with VA044 (0.73 g, 2.27 mmol) dissolved in water (202.9 g). A third flask was charged with water. The DMAm/TTC1 and solvent reservoirs were kept at room temperature in air. The initiator reservoir was kept refrigerated at 5 °C for the duration of the experiments to ensure that the initiator did not decompose overnight. 15 training experiments were generated and conducted using a Latin Hyper Cube algorithm.

Table S 2. *Reaction parameter space explored in the TSEMO of the dimethylacrylamide optimisations.*

Input	Lower Limit	Upper Limit	Variance Limit
Temperature (°C)	50	80	2
Residence Time (min)	5	20	1
[I]:[CTA]	0.04	0.09	0.005

Table S 3. *Rate parameters used for model-informed platform for the polymerisation of dimethylacrylamide.*

	$E_a (kJmol^{-1})$	A	Ref.
k_d	108.20	1.28×10^{12}	[8]
k_p	14.10	1.10×10^{7}	[9]
k_a	0.00	5.66×10^{6}	[10]
k_t	25.00	2.00×10^{11}	[11]
Other parameters			
f	0.60		
α_s	0.40		

α_l	0.18
L_c	50
K	300
k_{ct}	$0.25k_t$

Data

Table S 4. *Dataset for the black box optimisation of dimethylacrylamide, the grey data corresponds to the Latin hypercube sampling data.*

Experiment #	[I]:[CTA]	Temperature(°C)	Residence Time (min)	Đ	α (%)
1	0.060	52	18	1.151	78.5
2	0.075	54	15	1.199	86.1
3	0.050	54	6	2.000	10.9
4	0.065	58	13	1.213	92.9
5	0.080	58	20	1.236	99.4
6	0.070	60	7	1.164	76.9
7	0.055	64	9	1.182	90.9
8	0.045	66	11	1.242	96.6
9	0.090	68	8	1.229	100.4
10	0.060	70	18	1.297	101.4
11	0.065	70	11	1.263	100.5
12	0.050	72	12	1.243	101.5
13	0.080	74	14	1.309	101.7
14	0.040	78	8	1.282	100.1
15	0.085	78	17	1.348	101.3
16	0.085	62	15	1.243	102.2

17	0.060	64	11	1.234	97.3
18	0.090	76	10	1.367	100.6
19	0.055	76	16	1.333	102
20	0.060	80	19	1.369	100.7
21	0.060	64	15	1.232	101.5
22	0.040	50	10	2.000	0.1
23	0.080	58	6	1.154	64.3
24	0.085	70	11	1.372	100.1
25	0.075	72	8	1.328	98.5
26	0.040	72	18	1.406	102.8
27	0.090	64	6	1.176	94.0
28	0.060	72	5	1.243	97.5

Table S 5. *Dataset for the model-informed optimisation of dimethylacrylamide. Latin hypercube sampling data is shaded grey and the first iteration of TSEMO shaded blue.*

Experiment #	[I]:[CTA]	Temperature(°C)	Residence Time (min)	Đ	α (%)
1	0.060	52	18	1.196	90.6
2	0.075	54	15	1.197	92.2
3	0.050	54	6	1.171	44.0
4	0.065	58	13	1.198	93.2
5	0.080	58	20	1.196	99.6
6	0.070	60	7	1.208	77.0
7	0.055	64	9	1.202	91.0
8	0.045	66	11	1.200	96.0
9	0.090	68	8	1.202	98.0
10	0.060	70	18	1.201	100.0

11	0.065	70	11	1.202	99.6
12	0.050	72	12	1.202	99.8
13	0.080	74	14	1.205	100.0
14	0.040	78	8	1.206	99.3
15	0.085	78	17	1.209	100.0
1	0.080	50	16	1.185	77.7
2	0.060	56	8	1.142	58.1
3	0.060	56	20	1.239	102.8
4	0.040	78	11	1.315	101.1
5	0.060	56	6	1.138	26.1
6	0.055	56	11	1.164	68.0
7	0.075	68	19	1.398	104.6
8	0.065	60	20	1.306	101.8
9	0.050	50	14	2.000	0.60
10	0.040	58	18	1.194	89.8
11	0.065	54	18	1.184	85.4
12	0.085	74	17	1.383	100.4
13	0.080	80	20	1.481	99.5
14	0.070	56	18	1.197	92.4
15	0.065	78	11	1.318	100.2

Performance Metric

Hypervolume is used by TSEMO as a performance criteria to identify the amount of objective space that has been explored relative to the worst possible set of outcomes, i.e., Conversion of 100% and a Đ of 2. Here, the hypervolume has been calculated to compare both the model-informed and blackbox optimisations for their ability to maximise the volume of objective space. As the main TSEMO algorithm on builds GPs on real experimental data the initial in silico LHC

experiments are not counted in these calculations, the initial 5 experiments for the model-informed platform were considered the training experiments. The plots showing the hypervolume improvement with respect to each experiment can be seen in Figure S 11.

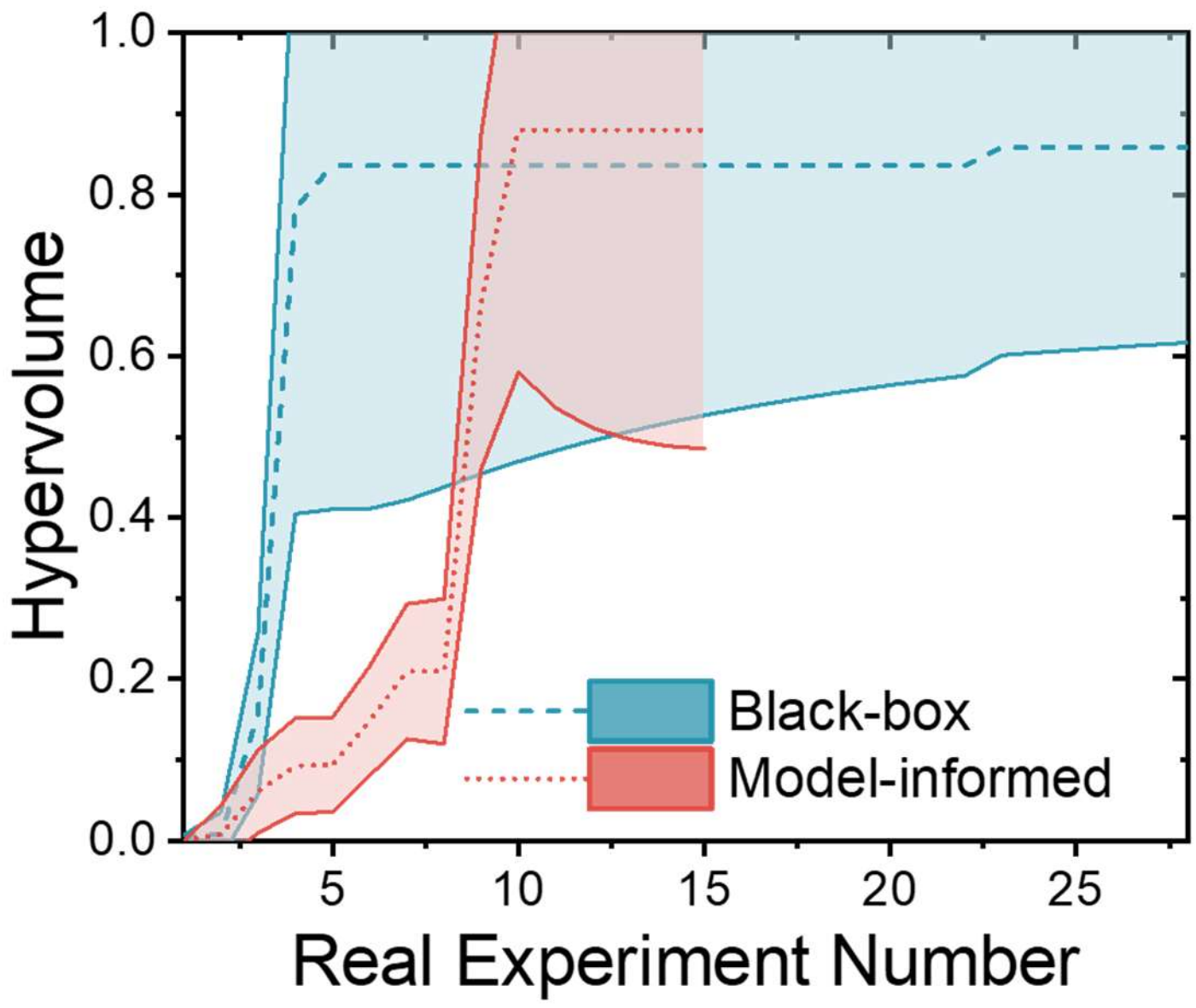

Figure S 11. *Hypervolume convergence plots comparing the real experimental data collected from the fully experimental "black box" platform (blue data) to the model-informed platform (red data) for the RAFT polymerisation of dimethylacrylamide.*

Figure S 11 shows the model-informed platform to identify more of the objective space within the initial model-informed solutions determined by the initial TSEMO iteration. The amount of uncertaintly in the performance reduces over time as more experiments are conducted. Greater error in the model-informed platofrm can be accounted for as subsequent Iterations of TSEMO are based off 10 fewer physical experiments than the black-box optimisation.

4. RAFT Polymerisation of n-butyl acrylate

To a solution of nBuA (200 g, 1.56 mol) in 1,4-dioxane (134.7 g), TTC1 (1.86 g, 70 mmol) was added. A separate flask was charged with AIBN (0.289g , 1.76 mmol) dissolved in 1,4-dioxane (202.1 g). A third flask was charged with water. These were kept at room temperature, and the solutions were not degassed under an atmosphere of nitrogen. 15 training experiments were generated and conducted using a Latin Hyper Cube algorithm.

Table S 6. *Reaction parameter space limits for the TSEMO of n-butyl acrylate optimisations.*

Input	Lower Limit	Upper Limit	Variance Limit
Temperature (°C)	70	100	2
Residence Time (min)	5	20	1
[I]:[CTA]	0.040	0.090	0.005

Table S 7. *Rate parameters used for model-informed platform for the polymerisation of butylacrylate.*

	$E_a (kJmol^{-1})$	A	Ref.
k_d	132.20	1.28×10^{12}	[8]
k_p	17.90	2.24×10^{7}	[12]
$k_a *$	0.00	6.0×10^{6}	[10,13]
k_t	10.00	1.30×10^{10}	[14]
Other parameters			
f	0.42		
α_s	0.56		
α_l	0.16		
L_c	33		

K	100
k_{ct}	$0.25k_t$

Data

Table S 8. *Dataset for the black box optimisation of butyl acrylate, the shaded grey data corresponds to the Latin hypercube sampling data.*

Experiment #	[I]:[CTA]	Temperature(°C)	Residence Time (min)	Đ	α (%)
1	0.060	72	18	1.431	35.1
2	0.075	74	15	1.412	30.5
3	0.050	74	6	2.000	6.70
4	0.065	78	13	1.266	32.1
5	0.080	78	20	1.203	47.4
6	0.070	80	7	1.089	18.9
7	0.055	84	9	1.205	33.1
8	0.045	86	11	1.230	36.7
9	0.090	88	8	1.310	42.8
10	0.060	90	18	1.376	63.1
11	0.065	90	11	1.340	50.3
12	0.050	92	12	1.325	51.6
13	0.080	94	14	1.421	64.0
14	0.040	98	8	1.361	50.1
15	0.085	98	17	1.997	73.7
16	0.045	90	20	1.326	56.7
17	0.045	84	20	1.335	50

18	0.060	100	14	1.430	67.8
19	0.040	100	19	1.371	65.6
20	0.060	96	17	1.339	64.3
21	0.045	98	10	1.378	54.7
22	0.085	86	16	1.441	54.6
23	0.090	86	18	1.376	64.8
24	0.080	88	20	1.409	70.0
25	0.045	88	18	1.337	54.2
26	0.055	96	20	1.451	68.5
27	0.090	90	16	1.484	57.9
28	0.065	96	15	1.422	67.9
29	0.055	92	16	1.365	59.9
30	0.090	84	20	1.390	60.2
31	0.065	94	20	1.416	70.1
32	0.065	98	18	1.393	72.2
33	0.060	82	18	1.384	51.6
34	0.070	80	19	1.329	50.2
35	0.085	76	20	1.277	37.4
36	0.075	88	18	1.462	69.1
37	0.065	100	20	1.339	73.0
38	0.050	86	16	1.228	36.5
39	0.080	80	17	1.309	44.6
40	0.060	100	19	1.477	76.0
41	0.065	100	9	1.410	61.4

Table S 9. *Dataset for the model-informed optimisation of butyl acrylate. Latin hypercube sampling data is shaded grey and the first iteration of TSEMO shaded blue.*

Experiment #	[I]:[CTA]	Temperature(°C)	Residence Time (min)	Đ	α (%)
1	0.06	72	18	1.228	20.7
2	0.075	74	15	1.218	23.1
3	0.05	74	6	1.627	5.50
4	0.065	78	13	1.211	25.9
5	0.08	78	20	1.161	46.4
6	0.07	80	7	1.299	15.3
7	0.055	84	9	1.222	26.0
8	0.045	86	11	1.193	34.5
9	0.090	88	8	1.183	41.7
10	0.060	90	18	1.182	77.9
11	0.065	90	11	1.166	55.6
12	0.050	92	12	1.163	60.6
13	0.080	94	14	1.191	85.5
14	0.040	98	8	1.176	55.8
15	0.085	98	17	1.204	97.9
1	0.090	92	19	1.292	67.3
2	0.090	92	20	1.352	70.5
3	0.090	94	18	2.000	91.0
4	0.085	94	10	1.250	63.5
5	0.055	72	19	1.356	58.2
6	0.090	98	11	1.094	29.0
7	0.070	80	5	1.383	65.4
8	0.085	78	10	2.000	12.4
9	0.085	92	8	1.184	28.1
10	0.085	78	16	1.322	50.0

11	0.045	82	19	1.313	43.0
12	0.085	100	18	1.283	45.3
13	0.090	100	18	1.387	74.2
14	0.090	100	13	1.438	75.4
15	0.075	94	7	1.477	71.9
16	0.040	86	16	1.322	48.2
17	0.045	96	19	1.317	46.4
18	0.055	100	16	1.416	67.6
19	0.040	98	17	1.451	71.4
20	0.085	100	8	1.466	66.7
21	0.090	98	18	1.395	61.0
22	0.090	92	15	1.474	77.6
23	0.080	92	12	1.487	67.8
24	0.075	98	18	1.396	65.2

Performance Metric

The hypervolume improvement was also conducted for the polymerisation of n-butylacrylate which are illustrated in **Figure S 12**.

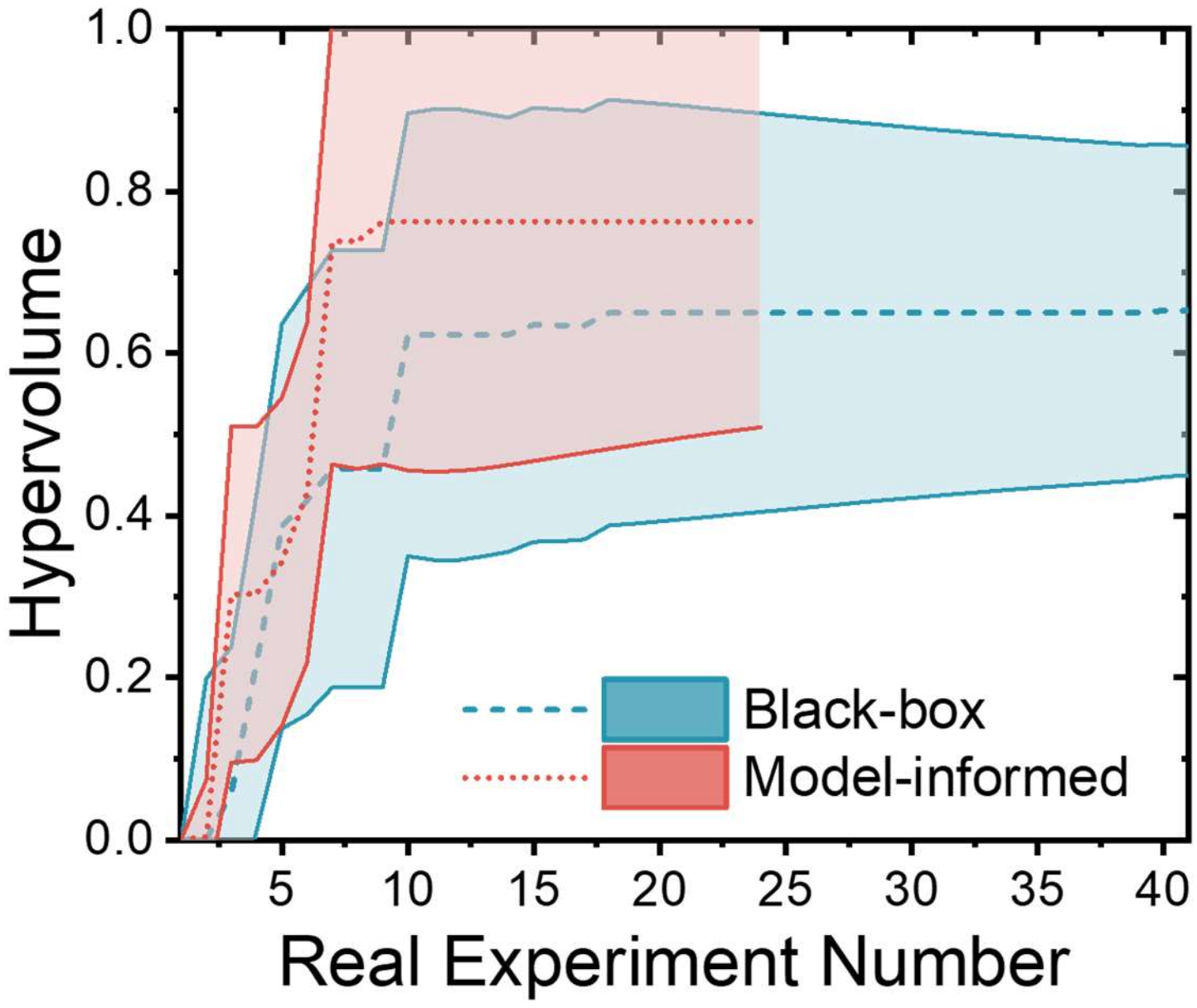

Figure S 12. *Hypervolume convergence plots comparing the data collected from the fully experimental "black-box" platform (blue data) to the model-informed platform (red data) for the RAFT polymerisaton of n-butylacrylate in dioxane.*

The model informed optimisation outperformed the blackbox optimisation based on the average hypervolume improvement, coinciding with the observations in **Figure S 11**, exploring more objective space in significantly fewer experiments. The high uncertainty bounds observed in the black-box platform can be accounted for by the chemistry in question involving many side reactions which resulted in noisy data. As the hypervolumes by experiment 10 are not significantly different, illustrated by the overlap in standard deviations, it is still beneficial to use the model-informed platform.

References

1 S. T. Knox, S. Parkinson, R. Stone and N. J. Warren, *Polym Chem*, 2019, **10**, 4774–4778.

2 E. Bradford, A. M. Schweidtmann and A. Lapkin, *Journal of Global Optimization*, 2018, **71**, 407–438.

3 A. D. Clayton, J. A. Manson, C. J. Taylor, T. W. Chamberlain, B. A. Taylor, G. Clemens and R. A. Bourne, *React Chem Eng*, 2019, **4**, 1545–1554.

4 P. Müller, A. D. Clayton, J. Manson, S. Riley, O. S. May, N. Govan, S. Notman, S. V. Ley, T. W. Chamberlain and R. A. Bourne, *React Chem Eng*, 2022, **7**, 987–993.

5 M. D. McKay, *Proceedings - Winter Simulation Conference*, 1992, 557–564.

6 C. Y. P. Wilding, S. T. Knox, R. A. Bourne and N. J. Warren, *Macromolecules*, 2023, **56**, 1581–1591.

7 A. R. Wang and S. Zhu, *J Polym Sci A Polym Chem*, 2003, **41**, 1553–1566.

8 J. Brandrup, E. H. Immergut and E. A. Grulk, *Polymer Handbook fouth edition*, 1999.

9 J. Schrooten, I. Lacík, M. Stach, P. Hesse and M. Buback, *Macromol Chem Phys*, 2013, **214**, 2283–2294.

10 G. Moad, E. Rizzardo and S. H. Thang, *Aust J Chem*, 2012, **65**, 985–1076.

11 A. Valdebenito and M. V. Encinas, *Polym Int*, 2010, **59**, 1246–1251.

12 A. P. Haehnel, B. Wenn, K. Kockler, T. Bantle, A. M. Misske, F. Fleischhaker, T. Junkers and C. Barner-Kowollik, *Macromol Rapid Commun*, 2014, **35**, 2029–2037.

13 J. J. Haven, N. Zaquen, M. Rubens and T. Junkers, *Macromol React Eng*, 2017, **11**, 1700016.

14 J. Barth, M. Buback, P. Hesse and T. Sergeeva, *Macromolecules*, 2010, **43**, 4023–4031.
